# Off the wall: The rhyme and reason of *Neurospora crassa* hyphal morphogenesis

**DOI:** 10.1016/j.tcsw.2019.100020

**Published:** 2019-03-08

**Authors:** Jorge Verdín, Eddy Sánchez-León, Adriana M. Rico-Ramírez, Leonora Martínez-Núñez, Rosa A. Fajardo-Somera, Meritxell Riquelme

**Affiliations:** a Industrial Biotechnology, CIATEJ-Jalisco State Scientific Research and Technology Assistance Center, Mexico National Council for Science and Technology, Zapopan, Jalisco, Mexico; b Michael Smith Laboratories, University of British Columbia, Vancouver, British Columbia, Canada; c Department of Microbiology, Centro de Investigación Científica y de Educación Superior de Ensenada, CICESE Ensenada, Baja California, Mexico; d Department of Biochemistry and Molecular Pharmacology, University of Massachusetts Medical School, Worcester, MA, USA; e Karlsruhe Institute of Technology (KIT) South Campus, Institute for Applied Biosciences, Department of Microbiology, Karlsruhe, Germany

**Keywords:** CWI, cell wall integrity, CWP, cell wall proteins, CHS, chitin synthase, CLSM, confocal laser scanning microscopy, ER, endoplasmic reticulum, FRAP, fluorescence recovery after photobleaching, GSC, β-1,3-glucan synthase complex, BGT, β-1,3-glucan transferases, GH, glycosyl hydrolases, GPI, glycosylphosphatidylinositol, GFP, green fluorescent protein, GEF, guanine nucleotide exchange factor, MS, mass spectrometry, MT, microtubule, MMD, myosin-like motor domain, NEC, network of elongated cisternae, PM, plasma membrane, SPK, Spitzenkörper, TIRFM, total internal reflection fluorescence microscopy, TM, transmembrane, Cell wall, Tip growth, Spitzenkörper, Vesicles

## Abstract

The fungal cell wall building processes are the ultimate determinants of hyphal shape. In *Neurospora crassa* the main cell wall components, β-1,3-glucan and chitin, are synthesized by enzymes conveyed by specialized vesicles to the hyphal tip. These vesicles follow different secretory routes, which are delicately coordinated by cargo-specific Rab GTPases until their accumulation at the Spitzenkörper. From there, the exocyst mediates the docking of secretory vesicles to the plasma membrane, where they ultimately get fused. Although significant progress has been done on the cellular mechanisms that carry cell wall synthesizing enzymes from the endoplasmic reticulum to hyphal tips, a lot of information is still missing. Here, the current knowledge on *N. crassa* cell wall composition and biosynthesis is presented with an emphasis on the underlying molecular and cellular secretory processes.

## Highlights

• Chitin and β-1,3-glucan synthases are transported separately in chitosomes and macrovesicles.
• Chitin synthases occupy the core of the SPK; β-1,3-glucan synthases the outer layer.
• CHS-4 arrival to the SPK and septa is CSE-7 dependent.
• Rabs YPT-1 and YPT-31 localization at the SPK mimics that of chitosomes and macrovesicles.
• The exocyst acts as a tether between the SPK outer layer vesicles and the apical PM.

## 1 Introduction

The filamentous fungus *Neurospora crassa* has been used for decades as a model system to investigate the genetic basis of phenotypical traits, metabolic pathways, circadian rhythms, and gene silencing, among other biological processes (De Terra and Tatum, 1961, Hammond, 2017, Mahadevan and Tatum, 1965, Roche et al., 2014, Seiler and Plamann, 2003, Selker et al., 1987). Most recently, *N. crassa* has become also an important model microorganism to investigate cellular processes such as morphogenesis, cell polarization and cell-cell fusion (Daskalov et al., 2017, Lichius et al., 2012, Riquelme et al., 2011). As in other filamentous fungi, *N. crassa* hyphae have a cell wall that allows them to deal and interact with the surrounding environment, and that determines their own growth and shape. While the composition of *Neurospora*’s cell wall is well known, complete understanding of the underlying molecular and cellular mechanisms that contribute to its synthesis, assembly and remodeling is lacking. The extraordinary advancement of live cell imaging technologies together with the tractable genetic manipulation of *Neurospora*’s cells, have allowed the study of the fate and mode of operation of the cell wall synthesis machinery, including organelles, associated cytoskeleton and regulatory components involved in their secretion.

This review focuses on the subcellular mechanisms that lead to cell wall assembly and remodeling with a special emphasis in apical processes. First, we summarize the current knowledge on *Neurospora* cell wall composition and structure and discuss the proposed models for cell wall synthesis. Next, the review concentrates on the cellular processes involved in the intracellular trafficking and sorting of *Neurospora*’s cell wall building nanomachinery. A special emphasis is given to the composition and function of the Spitzenkörper (SPK), the apical body that serves as the main choreographer of tip growth and hyphal morphogenesis.

## 2 *Neurospora crassa* cell wall composition, structure and assembly: not just another brick in the wall

### 2.1 Composition and structure

**Fig. 1:**
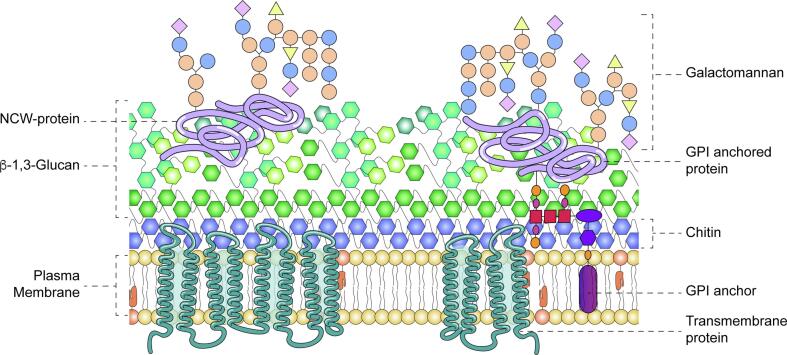
Graphic representation of the cell wall structure of *N. crassa.* The cell wall is illustrated as a matrix of superimposed layers of polysaccharides. A compact layer of chitin fibrils is the closest to the plasma membrane, followed by a thicker layer of mixed β-1,3 and other glucans. The latter acts as scaffold for galactomannans and cell wall resident proteins, that may or may not have lost their GPI anchor to the plasma membrane. The lipid bilayer holds integral membrane proteins required for the synthesis of the cell wall polysaccharides. GPI, glycosylphosphatidylinositol; NCW, non-anchored cell wall protein.

The cell wall of *N. crassa* hyphae is a composite of superimposed layers (Fig. 1) (Mahadevan and Tatum, 1967). The innermost layer, the closest to the plasma membrane (PM), is an alkali-insoluble fibrillar skeleton containing primarily glucan (either lineal β-1,3-glucan or branched β-1,3-glucan with β-1,6 linkages at branching points) and a small percentage of chitin, whereas the outermost layer is an alkali-soluble amorphous cement containing cell wall proteins (CWP) covalently bound to the β-1,3-glucan either via the remnants of a GPI moiety or directly through an α-1,6 bond to the core of N-linked gel type polysaccharides (galactan and mannan) (Blumenthal and Roseman, 1957, Cardemil and Pincheira, 1979, Leal et al., 1996, Mahadevan and Tatum, 1965, Manocha and Colvin, 1967, Mélida et al., 2014, Nakajima et al., 1984). Conidial cell walls contain also α-1,3-glucan, a component that is not detected in cell walls of vegetative hyphae (Fu et al., 2014b). In other fungi, α-1,3-glucan is often found agglutinating chitin and β-1,3-glucan to protect their exposure to the external milieu (Fu et al., 2014b). Besides those main components, small amounts of glucuronic acid have been also detected in *N. crassa* cell walls; nevertheless, the exact nature and function of the glucuronic acid remains unknown (Bartnicki-Garcia, 1968, Cardemil and Pincheira, 1979, Mélida et al., 2014).

During the 1960s, the cell wall chemistry was considered a characteristic to systematically classify fungi since some monosaccharides were consistently present in the cell walls of defined fungal taxa: d-galactose and d-galactosamine (Ascomycetes), l-fucose (Mucoromycetes and Basidiomycetes), d-glucosamine (Mucoromycetes), and xylose (Basidiomycetes) (Bartnicki-Garcia, 1968). Earlier inferences made by cell wall biochemists have been confirmed by molecular phylogenetics and phylogenomics studies (Spatafora et al., 2017, Stajich, 2017). Recently, a high proportion of β-1,3 linked fucose-containing polysaccharides was found in two Mucoromycota species, *Phycomyces blakesleeanus* and *Rhizopus oryzae* (Mélida et al., 2014). Corresponding genes involved in fucose metabolism were found in this early divergent phylum, while they were absent in *N. crassa*. Moreover, it was found that both Mucoromycota species only harbor four of the seven CHSs classes (I, IV, V, VII) typically observed in Dikarya, and a new class (VIII, Division 2) that seems exclusive of the Mucoromycota. Nevertheless, contemporary biological systematics has ruled out cell wall composition as synapomorphy, as well as lysine biosynthesis and the presence of ergosterol in cell membranes, since such traits are neither exclusive nor always present in all members of the Kingdom Fungi (Richards et al., 2017).

The CWPs include a combination of glycosyl hydrolases such as chitinases, chitosanases, β-1,3-endoglucanases, β-1,6-endoglucanases, exoglucanases, mixed linked glucanases, and β-1,3-glucanosyltransferases (De Groot et al., 2005, Maddi et al., 2009, Maddi et al., 2012b). All these CWPs could have a role in cell wall remodeling, presumably a necessary process for hyphal growth and branching to ensue (see Section 4). As for other identified CWPs, very few of them have known associated functions. HAM-7 (NCU00881) is a cellular receptor that acts during cell anastomosis (Maddi et al., 2012a). ACW-2 (NCU00957), ACW-3 (NCU05667), and ACW-7 (NCU09133) are cell wall GPI-modified CWPs containing a Kre9 domain important for β-glucan assembly (Maddi et al., 2009). ACW-5 (NCU07776) and ACW-6 (NCU03530) contain CFEM domains rich in cysteines found in proteins involved in fungal pathogenesis (Maddi et al., 2009).

### 2.2 Cell wall assembly

The construction of the cell wall is the result of a series of finely orchestrated events (Bartnicki-Garcia and Lippman, 1969). Yet, an understanding of the mechanisms that take place during tip elongation, branching and spore formation is still limited. In vegetative hyphae, the synthesis and early assembly of the cell wall components occur at the tips (extension zone) as a consequence of a highly polarized secretory process (Bartnicki-Garcia and Lippman, 1969, Riquelme, 2013). The main structural polysaccharides (β-1,3-glucan and chitin) are synthesized at secretion sites (Hartland et al., 1994). In contrast, CWP (anchored and non-anchored) and polysaccharides of the amorphous layer are pre-synthesized intracellularly, presumably through the ER-to-Golgi secretory pathway, and incorporated into the cell wall (Brul et al., 1997).

**Fig. 2:**
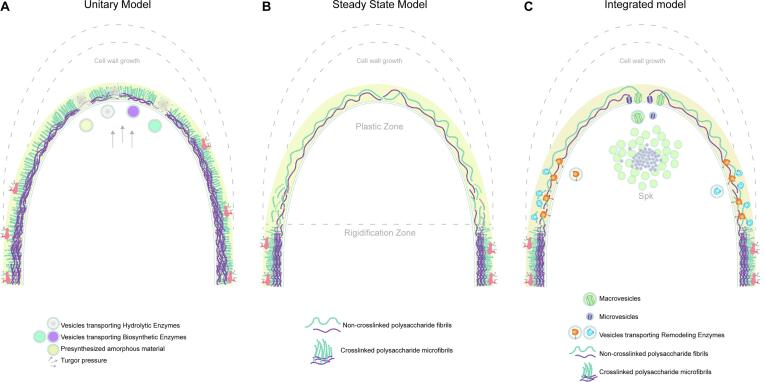
Proposed models for cell wall assembly. A. Unitary model of cell wall growth: an exocytosis gradient of vesicles at the apex, with a maximum at the most apical region and decreasing towards the subapex, would deliver both biosynthetic and hydrolytic enzymes. At the same time, the turgor pressure would expand the cell, allowing the incorporation into the plasma membrane of new vesicles containing synthesizing enzymes (Bartnicki-García, 1973). B. Steady state model: the newly synthesized cell wall fibrils at the growing hyphal tip are “plastic” (single chains of non-crosslinked polysaccharides), whereas at the base of the hyphal extension zone glucanosyltransferases would crosslink β-1,3-glucans and chitin resulting in a hardened cell wall (Vermeulen and Wessels, 1986). C. Integrated view: newly synthesized chitin and β-1,3-glucans chains are hydrolyzed by chitinases and β-1,3-glucanases, respectively, displaying new free ends available as substrate for cross-linking glucanosyltransferases that would interconnect amenable residues to harden the cell wall promoting its maturation behind the extension zone (Bartnicki-García, 1999).

Based on observations in a variety of fungi including yeasts, at least two models for cell wall synthesis have been proposed (Fig. 2). The unitary model of cell wall growth, the purpose of which was to explain hyphal shape generation, proposed that the cell wall construction during apical extension requires a delicate balance between secreted synthesizing enzymes and lytic enzymes (Fig. 2A) (Bartnicki-García, 1973). This model emerged upon observations in *Mucor rouxii* cells after chitin synthesis inhibition with polyoxin D, which resulted in impaired growth and spore germination, followed by cell tip bursting, right at the sites where chitin synthesis takes place (Bartnicki-Garcia and Lippman, 1969, Bartnicki-Garcia and Lippman, 1972a). These results supported the hypothesis that hyphal growing tips have cell wall lytic potential that must be gradually released and delicately coordinated with polysaccharide synthesis, which can be easily disturbed by external stimuli (Bartnicki-Garcia and Lippman, 1972b). A few years later, based on cell wall fractionations of pulse-chase labeled *Schizophyllum commune*, the steady-state model tried to explain the differential cell wall composition between the apex and the subapex (Fig. 2B). This other model suggested that cell wall material is assembled into the apex as non-fibrillar chains that become gradually crosslinked by glucanosyltransferases, leading to fibril crystallization at the subapex, which contributes to the hardening of the cell wall (Wessels, 1988). Hence, the steady-state model does not call for the need of plasticizing pre-existing assembled material, although it considers the action of enzymes at the subapex that transfer β-1–3 glucans to chitin, which rigidify the cell wall during hyphal morphogenesis. The steady-state model favors the mechanism for hyphal extension proposed by Robertson in 1959, which involved insertion of new wall material (primary wall) at the apex, and rigidification (secondary wall) of the newly formed wall at the base of the extension zone (Robertson, 1959, Robertson, 1968).

An integrated interpretation of the aforementioned cell wall growth models considers the simultaneous controlled action, in space and time, of biosynthetic enzymes, hydrolytic-loosening enzymes and rigidifying enzymes (Fig. 2C) (Bartnicki-García, 1999). The proposed integrated model hypothesizes that once synthesized, chitin and β-1,3-glucans chains are exposed to hydrolysis by chitinases and β-1,3-glucanases, respectively. The hydrolyzed polysaccharides display new free termini that serve as substrate for cross-linking enzymes that would interconnect amenable residues to harden the cell wall, promoting its maturation (Martínez-Nuñez and Riquelme, 2015). The presence of enzymes able to break down pre-existing polysaccharides at the extension zone suggested the malleability of the tip cell wall, which ultimately would allow the insertion of nascent cell wall polysaccharides (Martínez-Nuñez and Riquelme, 2015). Wall deformability would also be necessary in subapical areas of branch emergence (Mahadevan Pr Fau – Mahadkar and Mahadkar, 1970, Martínez-Nuñez and Riquelme, 2015). Moreover, in regions behind the extension zone, a maturation process takes place where further material, both fibrillar and amorphous, is added or existing material is cross-linked, generating a thicker “non extensible” wall (Trinci and Collinge, 1975) (Fig. 2C).

Interestingly, none of the models proposed how the amorphous material integrates into the cell wall or its role in shape generation and wall maturation. Knowledge of how cell wall synthesizing and remodeling enzymes accomplish their functions is limited. The following section summarizes the available information regarding cell wall synthesis enzymes in *N. crassa*.

## 3 The wall-building nanomachinery

### 3.1 β-1,3-glucan synthesis

β-1,3-glucan polymers are synthesized in *N. crassa* by the glucan synthase complex (GSC), constituted by a catalytic subunit, FKS-1 (NCU06871), and at least one regulatory subunit, RHO-1 (NCU01484). GS-1 (NCU04189; *cot-2*) is another protein important for β-1,3-glucan synthesis since suppression of *gs-1* impairs β-1,3-glucan synthase activity and cell wall formation (Enderlin and Selitrennikoff, 1994). Although its precise role remains unknown, biochemical evidence showed co-sedimentation of GS-1 with cell fractions containing β-1,3-glucan synthase activity, which suggested that it constitutes part of the GSC (Awald et al., 1993, Hrmova et al., 1989, Verdín et al., 2009).

**Fig. 3:**
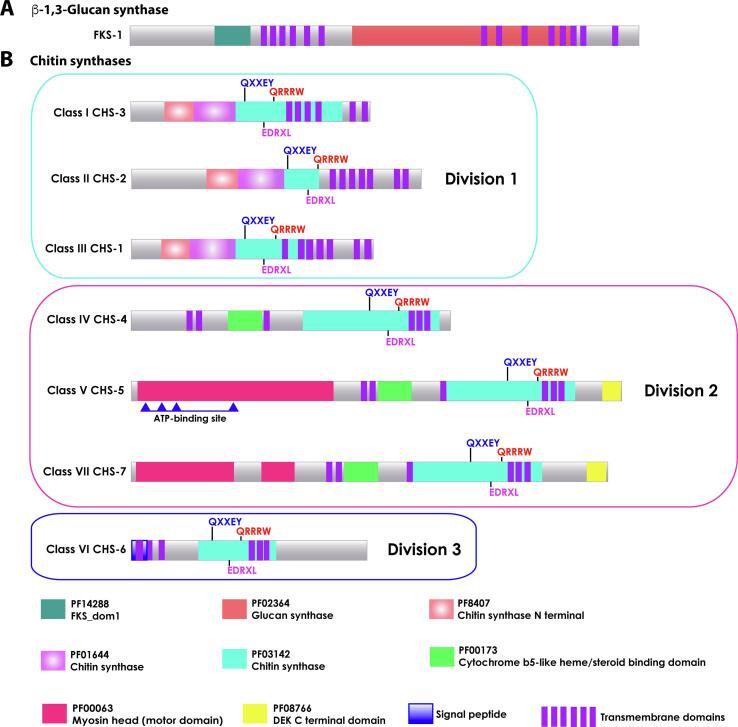
Illustrated domains of *N. crassa* cell wall synthases. A. β-1,3-glucan synthase FKS-1 (1955 aa; *fks-1,* NCU06871) is a transmembrane protein with fourteen transmembrane domains, one FKS_dom1 domain (PF14288), and one glucan synthase domain (PF02364). B. Chitin synthases are transmembrane proteins with three conserved sequence motifs QXXEY, EDRXL and QXRRW in the chitin synthase domain (PF03142). CHS-1, class III (917 aa; *chs-1,* NCU03611); CHS-2, class II (1097 aa; *chs-2,* NCU05239) and CHS-3, class I (903 aa; *chs-3,* NCU04251) are grouped in division 1, sharing the chitin synthase N-terminal domain (PF8407), and chitin synthase domain (PF01644). CHS-4, class IV (1235 aa; *chs-4*, NCU09324), CHS-5, class V (1874 aa; *chs-5*, NCU04352), and CHS-7, class VII (1819 aa; *chs-7*, NCU04350) are grouped in division 2, sharing the Cytochrome b5-like heme/steroid binding domain (PF00173). CHS-5 and CHS-7 additionally have a DEK C-terminal domain (PF08766), and a Myosin head motor domain (PF00063). CHS-6 (899 aa; *chs-6,* NCU05268) grouped in division 3, with a signal peptide at N-terminus. The domains were identified at the NCBI’s conserved domain database (CDD) and FungiDB (Marchler-Bauer et al., 2017, Basenko et al., 2018).

**Fig. 4:**
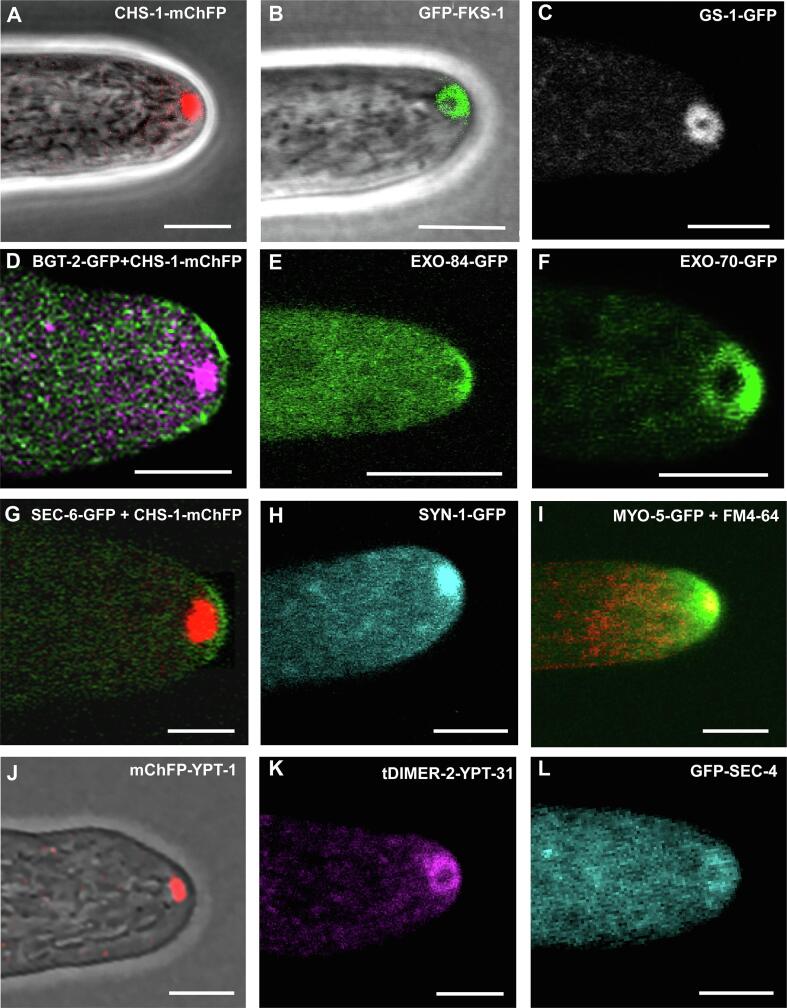
*N. crassa* hyphal apices showing the subcellular distribution of proteins related to: cell wall synthesis (A. CHS-1-mChFP, B. GFP^i^-FKS-1, C. GS-1-GFP), cell wall remodeling (D. BGT-2-GFP), vesicle tethering (E. EXO-84-GFP, F. EXO-70-GFP, G. SEC-6-GFP), vesicle fusion (H. SYN-1-GFP), vesicle transport (I. MYO-5-GFP stained with FM4-64) and vesicle trafficking (J. mChFP-YPT-1, K. tDIMER-2-YPT-31, L. GFP-SEC-4). BGT-2-GFP and SEC-6-GFP co-expressed with CHS-1-mChFP. Bars, 5 μm.

Fks1 is present in Ascomycota, Basidiomycota, Blastocladiomycota, Chytridiomycota, Cryptomycota, Glomeromycota and some *Incertae sedis* species (Richards et al., 2017). Similar to *Aspergillus fumigatus*, *N. crassa* contains a single copy of Fks1, which is required for cell viability and cell wall integrity (Lesage and Bussey, 2006, Mouyna et al., 2004, Schimoler-O'Rourke et al., 2003, Tentler et al., 1997, Thompson et al., 1999). *N. crassa* FKS-1 protein structure comprises a conserved large hydrophilic central domain flanked by six and eight transmembrane domains at its N- and C- terminus, respectively (Fig. 3A) (Sánchez-León and Riquelme, 2015). To determine the subcellular location and dynamics of *N. crassa* FKS-1, the GFP encoding gene was inserted in frame ∼200 amino acids upstream of the first transmembrane domain, close to the N-terminus of the protein (Sánchez-León and Riquelme, 2015). This strategy was followed since our previous attempts to C- and N-terminal tagging had proven unsuccessful. Live cell imaging of *N. crassa* GFP^i^-FKS-1 revealed its sub-cellular location at growing hyphal tips, specifically at the macrovesicular region of the SPK (outer layer), similarly to the spatial pattern observed for GS-1 (see below; Fig. 4B and C). FKS-1 was not only confined to the SPK, it was also detected at the foremost apical regions of the hyphal PM and slightly merging into the proximal limit of the subapical endocytic ring (Sánchez-León and Riquelme, 2015). This distribution led us to speculate that this region is where the macrovesicles containing FKS-1 might be discharged from the SPK and where FKS-1 is synthetically active before being inactivated. Although the precise mechanism of the synthetic activity of FKS-1 at the hyphal tip remains elusive, the apical localization of LRG-1 (RHO-1 GAP) and RGF-1 (RHO-1 GEF) were also indicative of the hyphal region where FKS-1 is presumably active (Richthammer et al., 2012, Vogt and Seiler, 2008).

In *Schizosaccharomyces pombe*, a fungus with no chitin in the cell wall, the FKS-1 orthologs Bgs1p and Bgs4p have been detected at septum formation sites (Cortes et al., 2005). By contrast, in *N. crassa* neither FKS-1 nor GS-1 have been detected at septa (Sánchez-León and Riquelme, 2015, Verdín et al., 2009), in agreement with earlier ultrastructural and biochemical studies showing chitin as the major polysaccharide of septa in *N. crassa* (Hunsley and Gooday, 1974).

Rho1 is a well-conserved protein across all fungal taxa that belongs to the family of Rho GTPases, molecular switches that through signal transduction pathways are involved in the regulation of different cellular processes, including cell wall integrity (CWI) maintenance (Levin, 2011). Both in *A. fumigatus* and in *S. cerevisiae*, Rho1 was identified as a component of the GSC (Beauvais et al., 2001, Levin, 2011). In *S. cerevisiae*, Rho1p regulates the synthesis of β-1,3-glucan in a GTP dependent manner and it seems that in *N. crassa* RHO-1 has a similar regulatory role in β-1,3-glucan synthesis (Lesage and Bussey, 2006, Qadota et al., 1996, Richthammer et al., 2012). Phenotypic analysis of *N. crassa rho-1* mutants revealed not only that RHO-1 is essential for cell viability but also that it is crucial for cell polarization and maintenance of CWI through the MAK-1 MAP kinase pathway via its direct interaction with Protein Kinase C 1 (PKC-1) (Richthammer et al., 2012, Vogt and Seiler, 2008). Although the interaction between FKS-1 and RHO-1 has not been confirmed in *N. crassa*, fluorescently tagged RHO-1 was also detected at the SPK. RGF-1 (NCU00668), a RHO-1 guanine nucleotide exchange factor (GEF), displayed a similar spatial distribution than FKS-1 (Richthammer et al., 2012, Sánchez-León and Riquelme, 2015). Whether RHO-1 and its regulator RGF-1 interact with FKS-1 or are required for its localization/activation needs to be determined. An appealing hypothesis is that RHO-1 might be strategically positioned at the SPK acting as a sensor ready to relay the signals to its CWI effector PKC-1 under cell wall stress conditions, as it has been observed in *S. cerevisiae* (Levin, 2005).

GS-1 has orthologs only in the Ascomycota and the Basidiomycota, confirming that fungi from these phyla evolved specific cell wall synthesis machinery (Verdin et al., unpublished). The best-characterized *N. crassa* GS-1 ortholog is *S. cerevisiae* Knr4p/Smi1p (Hong et al., 1994). Both GS-1 and Knr4p/Smi1p share a well-structured globular core flanked by two N- and C-terminal intrinsically disordered arms with a high capacity to form protein-protein complexes (Durand et al., 2008, Verdín et al., 2009). The N-terminal arm is essential for Knr4p/Smi1p cellular localization and interaction with partner proteins (Dagkessamanskaia et al., 2010a, Dagkessamanskaia et al., 2010b). The unstructured arms of *N. crassa* GS-1 could also play similar roles, since the N-terminally mCherryFP-tagged version of GS-1 accumulated in the cytosol (Verdin et al., unpublished) and the C-terminally GFP-labeled version, localized to the outer SPK layer (Fig. 4C), impaired the hyphal growth rate (Verdín et al., 2009). Knr4p/Smi1p interacts with proteins involved in CWI, bud emergence, and cell polarity establishment (Basmaji et al., 2006, Lesage et al., 2004). Although Knr4p/Smi1p does not interact with Fks1p, both of them are part of the PKC1-SLT2 signaling cascade, where Knr4p/Smi1p physically interacts and coordinates the signaling activity of Slt2p (Basmaji et al., 2006, Martin-Yken et al., 2003, Martin-Yken et al., 2002).

### 3.2 Chitin synthesis

Chitin is a β-1,4-linked homopolymer of *N*-acetyl glucosamine (GlcNAc) residues synthesized by chitin synthases (CHSs), a family of enzymes that catalyze the transfer of GlcNAc from UDP-GlcNAc to the reducing end of a growing chitin chain (Glaser and Brown, 1957). CHSs are polytopic proteins containing multiple transmembrane (TM) spanning domains and three conserved sequence motifs QXXEY, EDRXL, and QXRRW (Coutinho et al., 2003, Ruiz-Herrera et al., 2002, Saxena et al., 1995). Additionally, they contain a conserved catalytic domain, PF03142 (Fig. 3B) (Ruiz-Herrera et al., 2002).

Up to 15 CHS-encoding genes have been found in the genomes of Dikarya fungi, although only one to seven CHS-encoding genes are usually present in the genomes of Ascomycota (Goncalves et al., 2016). More expanded CHSs families are found in Mucoromycota, Chytridiomycota and Blastocladiomycota fungi (15–38 *chs* genes) (Goncalves et al., 2016, Mélida et al., 2014). The *N. crassa* genome contains seven CHS encoding genes: *chs-1* (NCU03611)*, chs-2* (NCU05239)*, chs-3* (NCU04251)*, chs-4* (NCU09324)*, chs-5* (NCU04352)*, chs-6* (NCU05268)*,* and *chs-7* (NCU04350) (Borkovich et al., 2004, Fajardo-Somera et al., 2015, Riquelme and Bartnicki-García, 2008). According to their amino acidic sequences, CHSs are grouped into seven classes, and in three divisions (Fig. 3B) (Choquer et al., 2004, Mandel et al., 2006, Riquelme and Bartnicki-García, 2008, Sheng et al., 2013). *N. crassa* Division 1 CHS comprises CHS-1, -2 and -3 belonging to classes III, II and I, respectively. In addition to the conserved domain PF03142, CHSs belonging to division 1 contain also a PF08407 and PF01644 domains. Division 2 contains CHSs of classes IV, V and VII, all of them characterized by a cytochrome b5-binding type domain. A DEK domain is found at the C-terminus of CHS-5 and CHS-7 (classes V and VII, respectively), which present also a myosin-like motor domain (MMD) at their N-terminus. In CHS-5 and CHS-7, the MMD domain has an ATPase activity domain that belongs to the larger group of P-loop NTPases. In CHS-5, the ATPase activity domain bears a putative phosphorylation site, a purine-binding loop, switch I and switch II regions, a P-loop and a SH1 helix. In CHS-7 the ATPase activity domain is shorter, and lacks the sites described for CHS-5. In addition, they both lack the IQ motif, characteristic of MYO-5, involved in binding calmodulin-like light chains (Odronitz and Kollmar, 2008). In classes V and VII CHSs of *A. nidulans* and *U. maydis,* the N-terminal MMD has a 20% identity to the MMD of class V myosin (Takeshita et al., 2006, Weber et al., 2006), while *N. crassa* CHS-5 and CHS-7 show 25% and 20% identity with MYO-5, respectively. CHS-6, belonging to class VI, does not group with any other CHS and it is the only CHS with an N-terminal signal peptide (Fajardo-Somera et al., 2015).

All seven *N. crassa* CHSs localize at the core of the SPK (Fig. 4A) and developing septa, although with subtle distribution differences (Fajardo-Somera et al., 2015, Riquelme et al., 2007, Sánchez-León et al., 2011). These observations, in combination with immunoprecipitation assays followed by mass spectrometry (MS) analyses, suggested that at least CHS-1, CHS-4 and CHS-5 are transported in distinct chitosome populations (see Section 5) (Fajardo-Somera et al., 2015).

As mentioned above, chitin is synthesized *in situ* at sites of secretion and CHSs are therefore delivered to those sites in an inactive form. Some CHSs are zymogens whose activation requires a proteolytic processing (Duran et al., 1975, Leal-Morales et al., 1988, McMurrough and Bartnicki-Garcia, 1971, Ruiz-Herrera et al., 1975). To date, specific proteases involved in CHSs activation have not been identified. However, immunoprecipitation/MS assays of *N. crassa* CHS-1, CHS-4 and CHS-5 have revealed putative proteases, which could participate in their regulation (Fajardo-Somera et al., 2015).

## 4 DIY cell improvement: a step-by-step guide for wall remodeling

### 4.1 Loosening of the cell wall: role of chitinases and glucanases

The fungal cell wall must serve as armor while still pliable. It is hypothesized that some cell wall resident GH facilitate the breakage between and within polysaccharides allowing the cell wall remodeling during morphogenetic changes and developmental transitions. Chitinases, on the one hand, are hydrolytic enzymes that efficiently cleave the β-1,4 linkage of chitin, releasing oligomeric and dimeric (chitobiose) products. Fungal chitinases are classified within the GH-18 family. Chitobiose residues can be further converted to monomeric residues (GlcNAc) by β-*N*-acetylglucosaminidases (Horsch et al., 1997), which in fungi have only been described within the GH-20 family (Seidl, 2008). Chitinases can be further classified as endo-hydrolases that cleave chitin at random positions, or exo-hydrolases that release chitobiose from either end of the polymer (Horn et al., 2006). A number of enzymes from the GH-18 family contain a secretory signal peptide for translocation across the ER membrane and entry into the secretory pathway, and a GPI anchor signal, which might determine their residency at the PM and/or cell wall, as well as N- or O-linked glycosylation sites for oligosaccharide modifications (Bowman and Free, 2006). This is why GH-18 glycoside hydrolases are considered potential cell wall modifying enzymes. Filamentous ascomycetes have generally between 10 and 30 GH-18 chitinase genes (Ihrmark et al., 2010, Karlsson and Stenlid, 2008).

The *N. crassa* genome includes 12 genes that encode putative chitinases belonging to the GH-18 family (Karlsson and Stenlid, 2009, Mélida et al., 2014, Tzelepis et al., 2012). A functional analysis of chitinases from GH-18 and GH-20 families in *N. crassa* revealed that 10 of these genes are non-essential. However, deletion of the gene *chit-1* (NCU02184) resulted in reduced growth rate compared to the wild type (Tzelepis et al., 2012). Despite this evidence is not enough to claim involvement of CHIT-1 in cell wall remodeling, its N-terminal signal peptide and a predicted C-terminus GPI anchor motif, suggest its potential cell wall localization and a possible role in modifying resident chitin (Tzelepis et al., 2012). In addition, *N. crassa* CHIT-1 is 39% identical to the *S. cerevisiae* Cts1, an endochitinase involved in mother-daughter cell separation (King and Butler, 1998, Kuranda and Robbins, 1987, Kuranda and Robbins, 1991). *N. crassa* CHIT-1 shows 36% identity with ChiA from *Aspergillus nidulans*, a protein localized at conidial germinating tubes, at hyphal branching sites and hyphal tips (Yamazaki et al., 2008). Despite their extensive presence in fungal genomes, very little is known about the function of chitinases during polarized tip growth. While it has been suggested they could have a role in plasticizing the cell wall (Arroyo et al., 2016, Bartnicki-García, 1999), to date studies are either inconclusive (Gooday et al., 1992, Tzelepis et al., 2012) or demonstrate that they have no role in fungal morphogenesis (Alcazar-Fuoli et al, 2011).

Glucanases, on the other hand, catalyze the breakage of the α or β glycosidic bond between two glucose subunits (Davies and Henrissat, 1995). They can be classified as endo- or exo-depending on the site where they cut along the chain (Pitson et al., 1993). The *N. crassa* genome contains at least 38 proteins annotated as glucan-modifying enzymes distributed among different GH families (Mélida et al., 2014). The protein encoded by NCU06010 corresponds to a mutanase or α-1,3-glucanase from the GH-17 family, which has been found only to be expressed throughout the conidiation process (Ao et al., 2016). This is consistent with the presence of α-1,3-glucans exclusively in the cell wall of asexual spores; however, no phenotypical defect has been observed in deletion mutants (Fu et al., 2014a). Interestingly, deletion of the orthologous protein Agn1 in *S. pombe* leads to clumped cells that remained attached to each other by septum-edging material, which in *S. pombe* is known to be a combination of α and β-1,3-glucans (Dekker et al., 2004).

The annotated gene NCU07076 encodes a putative β-1,3-glucanase classified into the GH-81 family. There have been no specific studies on this protein in *N. crassa*; however, their homologs have been related to cell wall remodeling during budding and cell separation in *S. cerevisiae* and *C. albicans*, respectively (Baladron et al., 2002, Esteban et al., 2005), and endolysis of the cell wall during sporulation in *S. pombe* (Encinar del Dedo et al., 2009). Another example is the NCU03914 translation product, that corresponds to a non-characterized β-1,3-exoglucanase belonging to the GH-5 family. *S. pombe* Exg1p, Exg2p and Exg3p, orthologs of NCU03914, are secreted to the periplasmic space, GPI-PM bound, or remain cytoplasmic, respectively. Interestingly, overexpression of Exg2p resulted in increased accumulation of α and β-1,3-glucans at the cell poles and septum, but deletion of *exg* genes seemed dispensable during these events (Dueñas-Santero et al., 2010).

Recently, the putative *N. crassa* GPI-modified β-1,3-endoglucanases BGT-1 (NCU06381) and BGT-2 (NCU09175) were tagged with GFP and imaged in live hyphae (Martínez-Nuñez and Riquelme, 2015). Both BGT-1 and BGT-2 were found to accumulate at the hyphal apical PM immediately behind the apical pole (Fig. 4D). Furthermore, both enzymes concentrated at polarization sites that seemingly involve cell wall growth and remodeling, such as septum development, branching, cell fusion and conidiation. BGT-1 and BGT-2 belong to the GH-17 family. Strains in which *bgt-1* or *bgt-2* were deleted displayed only a very slight reduction in growth rate; however, single *bgt-2* and double *bgt-1::bgt-2* deletion mutants exhibited an increased resistance to the cell wall stressors Calcofluor White and Congo Red, indicating an altered cell wall architecture. In addition, these mutants displayed conidiation defects, suggesting a role for BGT-1 and BGT-2 on the re-arrangement of glucans at the conidiophore cell wall to allow conidial separation (Martínez-Nuñez and Riquelme, 2015).

### 4.2 Hardening the cell wall: cross-linking activity of glucanosyltransferases

The *N. crassa* cell wall is a mixture of interconnected and branched β-1,3-glucans, chitin and proteins. Glucanosyltransferases are responsible for this activity. Many β-1,3-glucanases are able to hydrolyze and further transfer the cleaved residues to a new polysaccharide chain, thus behaving as glucanosyltransferases as well. Homologous proteins of *N. crassa* BGT-1 and BGT-2 have been previously reported as β-1,3-endoglucanases with glucanosyltransferase activity (Cappellaro et al., 1998, Chaffin, 2008, Mrsa et al., 1993, Sarthy et al., 1997, Sestak et al., 2004). BGT-1 and BGT-2 show 64% and 47% identity with *A. fumigatus* Bgt2, a PM-GPI bound branching enzyme that hydrolyzes β-1,3-glucan and transfers the residues to a different chain of β-1,3-glucan via a β-1,6-linkage (Gastebois et al., 2010, Mouyna et al., 2013, Mouyna et al., 1998). Even when further biochemical evidence is required, the considerable identity with *A. fumigatus* proteins suggests a similar role for BGTs in *N. crassa*.

CWPs with a role in β-1,3-glucan remodeling belonging to the GH-72 family or GEL (for *glucan elongating* β-1,3-glucanosyltransferase), are crosslinking enzymes with predicted GPI signals that have shown an active role in cell wall organization (Ao and Free, 2017, Kamei et al., 2013, Mouyna et al., 2000a). GEL family members cleave an internal glycosidic linkage of the β-1,3-glucan chains and transfer the newly formed reducing end to the non-reducing end of another β-1,3-glucan molecule. This results in the elongation of the polymer creating multiple anchoring sites for mannoproteins, galactomannans, chitin, and reorganizing β-1,3-glucans in the cell wall. GEL family members have been identified in several fungal species such as *S. cerevisiae* (Gas1), *C. albicans* (Phrp), and *A. fumigatus* (*GEL1*) (Cabib et al., 2007, Kitagaki et al., 2002, Mouyna et al., 2000b; Mouyna et al., 2005, Muhlschlegel and Fonzi, 1997, Nakazawa et al., 1998, Ragni et al., 2007a, Ragni et al., 2007b, Rodríguez-Peña et al., 2000, Rolli et al., 2011, Rolli et al., 2010, Saporito-Irwin et al., 1995, Tougan et al., 2002). *N. crassa* has 5 genes encoding for GEL family members (Ao and Free, 2017): *gel-1* or *gas-5* (NCU01162), *gel-2* (NCU06781), *gel-3* (NCU08909), *gel-4* (NCU07253), and *gel-5* (NCU06850). There is no evidence of the biochemical activity of these proteins in the cell wall of *N. crassa*; however, studies on mutant strains lacking one or several of the *gel* genes suggest that they play differential roles. GEL-3 is constitutively expressed and, in combination with GEL-4 and GEL-2, seems to be directly involved in vegetative growth, while in combination with GEL-1, participates actively in aerial hyphae and conidia production. GEL-1 and GEL-4 also display an active role in cell wall remodeling in response to stress conditions (Ao and Free, 2017, Kamei et al., 2013). While the main putative activity of the GEL family of β-1,3-glucanosyltransferases is the incorporation of newly synthetized β-1,3-glucan into the wall, it has been claimed that they are also important for glycoprotein incorporation (Ao and Free, 2017).

The CRH protein family (from Congo Red hypersensitive) is a second family of crosslinking enzymes with an active role during remodeling of cell wall polymers and anchored to the PM via a GPI anchor. The members of this family, Crh1p, Crh2p and Crr1p in *S. cerevisiae,* are homologous to bacterial β-1,3/1,4-glucanases (Planas, 2000) and plant xyloglucan endotransglycosylases/hydrolases (XETs/XTHs) (Rose et al., 2002). They act at different developmental stages in yeast (Gomez-Esquer et al., 2004, Rodríguez-Peña et al., 2000) and are classified within the GH-16 family at the CAZy database (Cantarel et al., 2009). Crh1p and Crh2p are the transglycosidases responsible for the transfer of chitin chains to β-1–6-glucan and to β-1–3-glucan in *S. cerevisiae in vivo* and the crosslinks they generate are essential for the control of morphogenesis (Blanco et al., 2012, Cabib, 2009, Cabib et al., 2007). There are 13 proteins members of the GH-16 family codified in the *N. crassa* genome; from them only MWG-1, GH-16-7 (NCU05974), CRF-1, GH-16-11 (NCU09117), and GH-16-14 (NCU09672) share 46%, 48% and 47% identity, respectively, with *S. cerevisiae* Crh1p. There is no evidence that indicates the active role of these proteins in crosslinking chitin to β-1,6-glucan and to β-1,3-glucan in *N. crassa*; however, the Crh enzymes are exclusive to fungi and well conserved across fungal genomes. The functional redundancy they share could be essential to act only during specific developmental stages and cell wall remodeling of the fungus.

## 5 Behind the scenes to a successful destination: the path of the CW building machinery

### 5.1 Chitosome biogenesis, trafficking and regulation: a pressing need

**Fig. 5:**
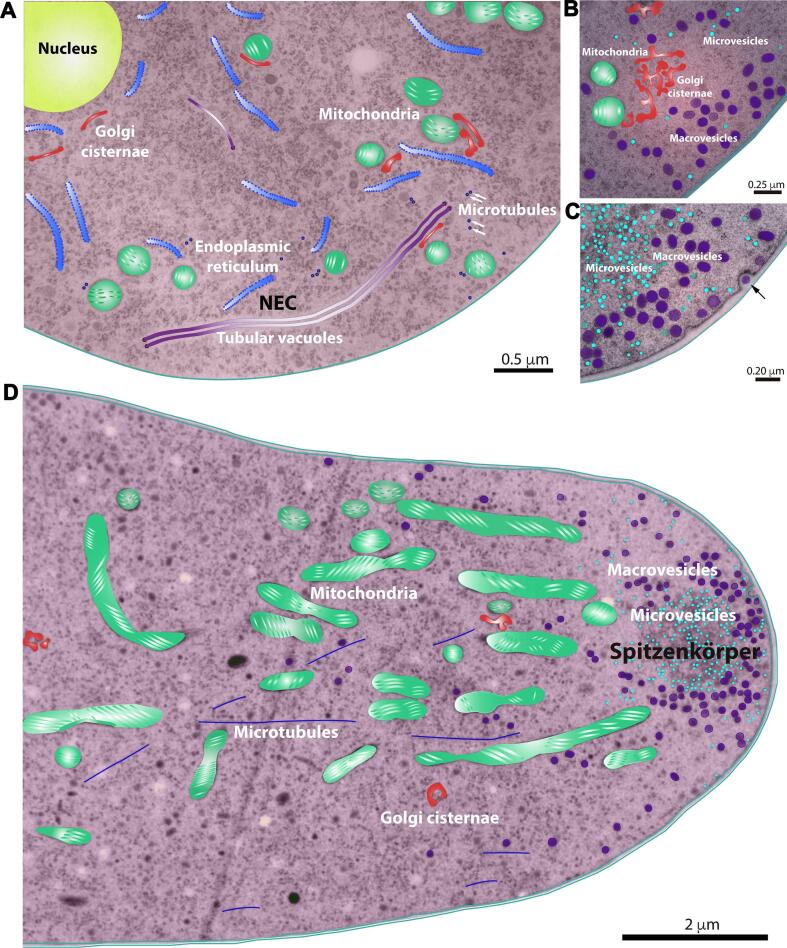
Colored transmission electron micrographs of thin sections of *N. crassa* hyphae. A. Cross section of a hypha showing the distribution and structure of different organelles; white arrows indicate microtubules. B. Cross section of a hypha showing the arrangement of the Golgi cisternae, the distribution of mitochondria, and some microvesicles and macrovesicles close to the plasma membrane. C. Longitudinal section of an apical region including the Spitzenkörper containing abundant microvesicles surrounded by macrovesicles. Also an exocytic event of one macrovesicle incorporated to the cell wall is observed (arrow). D. Longitudinal section of a hypha, including the proximal subapical region, showing microtubules running along the longitudinal axis of the hypha. At the tip the distribution and arrangement of microvesicles and macrovesicles can be easily seen. In this thin section, the mitochondria appear large and elongated. TEM images courtesy of R. Roberson, Arizona State University.

In their journey to the *N. crassa* hyphal tip, chitosomes first accumulate at the SPK core (Figs. 4A and 5B, C, D) and then presumably fuse with the cell PM to deposit CHSs at sites of cell wall expansion (Bartnicki-Garcia, 2006, Riquelme, 2013). These microvesicles have average diameters between 30 and 40 nm and a characteristic low buoyant density (1.13 g mL^−1^) (Martinez et al., 1989, Verdín et al., 2009).

For many years, it has been intriguing how the traffic of chitosomes toward the zones of active cell wall growth is organized and regulated in filamentous fungi. More than four decades ago, it was speculated that chitosomes could originate by self-assembly in the cytoplasm, could correspond to the intraluminal vesicles of multivesicular bodies, or could be derived from the endoplasmic reticulum (ER) (Bartnicki-Garcia, 1981). To elucidate the vesicular origin and traffic of chitosomes carrying CHSs in *N. crassa*, the effect of brefeldin A, an inhibitor of vesicular traffic between the ER and Golgi, was evaluated. Under the effect of the inhibitor at a concentration of 200 μg mL^−1^, CHS-1 continued to reach the core of the SPK (Sánchez-León et al., 2015), while CHS-4 did not, indicating that at least for some CHSs, such as CHS-4, transport occurs through the classical ER-to-Golgi secretory pathway (Rico-Ramírez et al., 2018).

Phosphorylation plays a role in the regulation of CHSs in *C. albicans*, where the correct localization and function of Chs3p depends on its phosphorylated state (Lenardon et al., 2010). Similarly, the phosphorylation state of Chs2p in *S. cerevisiae* determines its temporal and spatial localization (Chin et al., 2012, Teh et al., 2009, VerPlank and Li, 2005, Zhang et al., 2006). In *S. cerevisiae* Chs2p is directly phosphorylated by the cyclin-dependent kinase Cdk1p, and retained in the ER until the cell comes out of mitosis. Afterwards, Chs2p is dephosphorylated by the phosphatase Cdc14p. In a pulldown screen in *N. crassa*, the phosphatase PP1 (NCU00043) was identified as a putative CHS-4 interacting protein, and it was proposed that it could be acting as a regulatory protein with potential role in dephosphorylation of CHS-4 (Fajardo-Somera et al., 2015).

In *S. cerevisiae*, the vesicular traffic of Chs3p is well characterized*.* At the ER, Chs3p is palmitoylated by the action of palmitoyltransferase Pfa4p. This modification is necessary for Chs3p to achieve a competent conformation required for its ER exit (Lam et al., 2006). Moreover, the ER export cargo Chs7p (Starr et al., 2018) is responsible for directing the appropriate folding of Chs3p, avoiding its aggregation in the ER (Kota and Ljungdahl, 2005, Trilla et al., 1999). Chs3p is then transported to Golgi. From there, it can exit the Golgi through the formation of a complex called exomer, via an alternative exomer-independent pathway, or through an AP-1 dependent pathway (Starr et al., 2012). The exomer in *S. cerevisiae* consists of five proteins, which includes Chs5p and a family of four Chs5p-Arf1p binding proteins named ChAPs (Sanchatjate and Schekman, 2006, Trautwein et al., 2006, Wang et al., 2006). At the mother- bud neck septation sites, Chs4p and Bni4p mediate Chs3p activation and retention (DeMarini et al., 1997, Kozubowski et al., 2003, Ono et al., 2000, Sanz et al., 2004).

In *N. crassa*, genes encoding orthologs of all the proteins involved in the vesicular trafficking of Chs3p in *S. cerevisiae* have been identified. For Pfa4p (YOL003C) two orthologs were identified: palmitoyltransferase PFA-4 (NCU02118) and palmitoyltransferase PFA-3 (NCU01267). For Chs7p (YHR142W), two orthologous proteins were identified; CSE-7, a “chitin synthase export chaperone” (NCU05720), and a “hypothetical protein” (NCU01814). For the five components of the exomer complex, only two orthologous proteins were identified; CBS-5, a “chitin 5 biosynthesis protein” (NCU07435) ortholog for Chs5p (YLR330W), and BUD-7, a “Bud site selection protein” (NCU04511) ortholog for the four ChAPs (YJL099W, YMR237W, YKR027W, YOR299W). For Chs4p (YBL061C) three orthologs were identified: chitin synthase activator CSA-1 (NCU09322), chitin synthase activator CSA-2 (NCU02592), and chitin synthase regulator CSR-3 (NCU02351). From those, CSA-1 was found as an interacting partner of CHS-4, the ortholog of *S. cerevisiae* Chs3p (Fajardo-Somera et al., 2015). For Bni4p (YNL233W) an ortholog hypothetical protein (NCU00064) was identified.

Recently, in *N. crassa*, the role of CSE-7 on the secretory traffic of CHS-4 was evaluated (Rico-Ramírez et al., 2018). In a *Δcse-7* mutant background, CHS-4 arrival to the septum and to the core of the SPK was disrupted, and it instead accumulated in an endomembranous system distributed along the cytoplasm. The complementation of the mutation with a copy of the *cse-7* gene restored the location of the CHS-4 in zones of active growth, corroborating the role of CSE-7 as an ER receptor cargo for CHS-4 (Rico-Ramírez et al., 2018).

Fluorescently tagged CSE-7 was found in a network of elongated cisternae (NEC) similar to the compartments where CHS-4-GFP was retained in the mutant background for *Δcse-7* (Rico-Ramírez et al., 2018). Unexpectedly, CSE-7 appeared also at septa, as well as at the core of the SPK. For several decades *S. cerevisiae* Chs7p was thought to be a Chs3p chaperone confined to the ER (Trilla et al., 1999). However, recent studies have shown that Chs7p leaves the ER and apparently travels in the same vesicles that transport Chs3p to the cell surface, where it promotes Chs3p activity (Dharwada et al., 2018). These results agree with the presence of CSE-7 at the SPK in *N. crassa*, suggesting that CSE-7 could have an additional function at the apex (Rico-Ramírez et al., 2018).

*S. cerevisiae* Δ*CHS7* and *C. albicans chs7*Δ null mutants showed reduced levels of chitin content as well as decreased chitin synthase activity (Sanz et al., 2005, Trilla et al., 1999). In *N. crassa*, the Δ*cse-7* and *Δchs-4* mutants did not show any defects in growth rate, colony aberrant phenotype, or hyphal morphology when compared to the parental and wild type strains (Herold and Yarden, 2016, Rico-Ramírez et al., 2018). In contrast, Δ*chs-1*, Δ*chs-3*, Δ*chs-6,* and Δ*chs-7* single mutants, as well as the Δ*chs-1;* Δ*chs-3* double mutant showed a considerable decrease in growth rate and a disturbed phenotype (Fajardo-Somera et al., 2015). The lack of a mutant phenotype in *N. crassa* Δ*cse-7* suggested that CSE-7 does not have a role in the secretory pathway of CHS-1, -3, -6, and -7.

Extensive work is needed to identify the putative proteins involved in the secretory route of other CHSs in *N. crassa*. SEC-14 cytosolic factor (NCU02263) identified among the putative CHS-4 and CHS-5 interacting proteins could be potentially involved in the traffic of chitosomes (Fajardo-Somera et al., 2015). The homologs of Sec14p (YMR079W) in *S. cerevisiae* is involved in regulating the transfer of phosphatidylinositol (PtdIns) and phosphatidylcholine (PtdCho) and protein secretion (Bankaitis et al., 1990, Schnabl et al., 2003, Szolderits et al., 1989).

### 5.2 The endomembranous system: an intricate collection of tubules and cisternae.

The endomembranous system of *N. crassa* is a highly dynamic and complex network of elongated cisternae (NEC) that extends throughout the cytoplasm from region III (30–40 µM behind the apex) to distal regions of the hyphae. It comprises the so-called tubular vacuoles and the ER (Fig. 5A) (Rico-Ramírez et al., 2018).

In *S. cerevisiae*, the ER is described as a peripheral ER organized as an interconnected tubules and as perinuclear ER (Prinz et al., 2000). In *A. nidulans* and *Ustilago maydis* the ER appears as peripheral or cortical strands and nuclear envelopes (Markina-Iñarrairaegui et al., 2013, Wedlich-Söldner et al., 2002).

As mentioned above, CSE-7, the putative ER receptor for CHS-4, was localized in a highly dynamic NEC in close proximity to some nuclei but not exactly circling them. Two different vacuolar markers, the VMA-1 (ATP-ase vacuolar) and the dye Oregon Green 488 carboxylic acid (carboxy-DFFDA) (Bowman et al., 2015, Cole et al., 2000, Hickey et al., 2004), and two ER markers, NCA-1 and SEC-63 (Bowman et al., 2009, Rico-Ramírez et al., 2018), partially co-localized with CSE-7 at the NEC (Rico-Ramírez et al., 2018).

Transmission electron microscopy identified rough ER (RER) sheets abundantly distributed in subapical regions of the hyphae; nevertheless, smooth ER could not be detected. The RER was not observed in the vicinity of the nuclear envelope. The vacuoles appeared as extensive sheet-like cisternae with electron-transparent lumen, edges coated with an electron dense fibrillar material and tube-like extensions with electron-dense lumen containing heterogeneous inclusions, these two vacuolar morphologies appeared connected (Rico-Ramírez et al., 2018).

### 5.3 The secretion of β-1,3-glucan synthase.

Collectively, all the evidence gathered for *N. crassa* suggested that the GSC might be transported towards the hyphal tip in macrovesicles (Fig. 5B–D), a population of carriers different than the chitosomes (microvesicles, Fig. 5B–D; see previous section; Roberson et al., 2012, Sánchez-León and Riquelme, 2015, Verdín et al., 2009). Fluorescent recovery after photobleaching (FRAP) allowed analysis of the dynamics of the carriers containing the GFP tagged version of FKS-1. Fluorescence appeared at the immediate layers surrounding the core of the SPK and extended progressively to the most outer layers. Quantitative analysis of these FRAP experiments revealed significant differences between the half-time recovery values of FKS-1 and GS-1. These differences might indicate the type of association of these proteins with the macrovesicles. Whereas GS-1 transiently interacts with the vesicles, FKS-1 might be embedded in the vesicular membranes.

These observations contrast with what has been described for *U. maydis* where Msc1p, a class V CHS with an N-terminal MMD, is co-delivered to the apical PM together with Gsc1p (homolog of Fks1) or Chs6p (CHS class VII), suggesting one single population of secretory vesicles carrying cell wall synthesizing enzymes (Schuster et al., 2016). Electron micrographs of *U. maydis* filamentous cells indicate the presence of a few, similarly sized, vesicles in the apical region (Lehmler et al., 1997), which would explain the joint delivery of Gsc1p, Chs6p and Msc1p. More recently, it has been shown that the SPK of *C. albicans* contains also homogenously sized secretory vesicles (Weiner et al. 2019), suggesting that co-delivery of cell wall synthesizing enzymes could also occur in this and other fungal species. However, as cellular and biochemical evidence from *N. crassa* and other species indicate (Fajardo-Somera et al., 2015, Riquelme et al., 2014, Sánchez-León et al., 2015, Schultzhaus et al., 2015, Verdín et al., 2009), co-delivery is not a general secretion mechanism of cell wall synthesizing enzymes*.*

The differences observed in the organization of the vesicular conveyors of cell wall synthesizing enzymes in fungal species extend to their spatial organization at the apex, where they arrange in a diverse number of configurations besides the classical round SPK. Köhli et al (2008) established a correlation between the distribution of the apical vesicles (crescent shape, round SPK) and the hyphal growth rate of *Ashbya gossypii*. More recently, Dee et al. (2015) suggested that the distribution pattern of the apical vesicles is a specific trait of the major fungal phyla. Given the diversity of the fungal species they analyzed, which includes a diversity of growth rates, it is hard to conclude those patterns are the exclusive solution to a kinetic secretory necessity as Köhli et al suggested.

### 5.4 Rab GTPases: orchestrating the journey of secretory vesicles

Different laboratories have undertaken the challenge of elucidating the process by which the secretory vesicles are generated and transported to specific cell regions (Riquelme et al., 2018). The pioneering research in *S. cerevisiae* uncovered the mechanistic role of many of the regulatory molecules involved in the traffic of secretory vesicles (Bonifacino and Glick, 2004). In contrast to budding yeast, which experience transient polarized growth, the growth of fungal hyphae occurs continuously at a very high rate at their tips (Riquelme et al., 2016). This suggests important divergences of mechanisms of vesicle traffic between filamentous and yeast fungal forms.

**Fig. 6:**
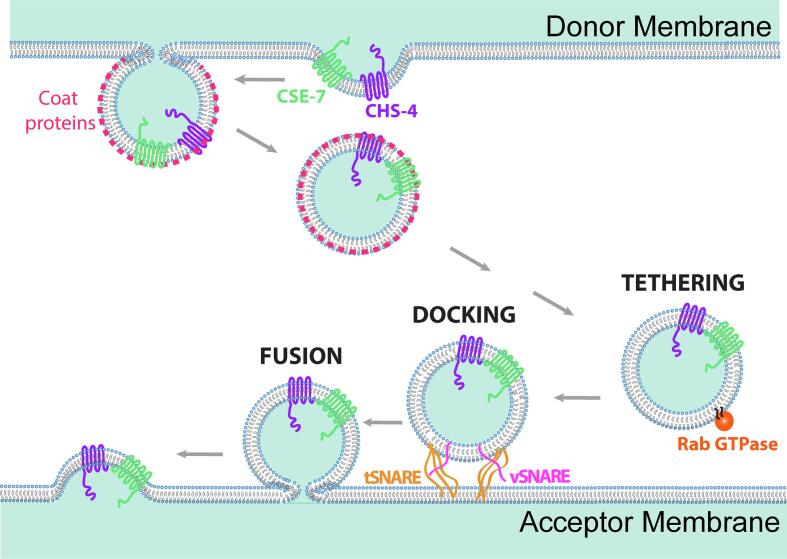
Graphic representation of the formation and exocytosis of vesicles. At the donor membrane a vesicle containing a cargo protein with transmembrane domains and a cargo adaptor/receptor protein also with transmembrane domains buds off. Concomitantly, coat proteins surround the vesicle, which is being transported with the assistance of the cytoskeleton to the acceptor membrane. Once the vesicle reaches the proximity of an acceptor membrane a tethering process coordinated by Rab GTPases, brings vesicle close to the acceptor membrane. The final step of the secretory process includes the docking of the vesicle to the target membrane, a process facilitated by SNARE proteins.

Vesicle secretion in all eukaryotic cells is orchestrated by the coordinated activity of small GTPases from the Rab family, protein coats, molecular motors, tethering factors and SNAREs (SNAP (Soluble NSF – N-ethylmaleimide-sensitive factor – Attachment Protein) Receptor) (Fig. 6) (Grosshans et al., 2006). Secretory proteins and their adaptors/receptors, recruited at specific foci of the donor membrane organelle, are enclosed in COPII-coated vesicles that initially bud off on their way towards the acceptor membrane (Barlowe and Miller 2013). The traffic of these secretory vesicles, assisted by molecular motors and the cytoskeleton, is finely coordinated by the action of Rab GTPases that act as signaling molecules ensuring the arrival of the vesicles to the specific downstream compartment (Jin et al., 2011, Herve and Bourmeyster, 2018). Tethering and fusion of the vesicles with the compartments are facilitated by the complex interplay between Rabs, monomeric and multimeric tethering proteins, and SNAREs, which facilitate the final delivery step of the vesicle cargoes (Segev et al., 1988, Sogaard et al., 1994, Nakajima et al., 1991). The activity of Rab proteins oscillates depending on the form of guanosine nucleosides (i.e. GDP or GTP) they are bound to. GEFs and Guanine Activating Proteins (GAPs) are implicated in the exchange of GDP for GTP and the hydrolysis of GTP to GDP, respectively. When bound to GTP, Rab proteins undergo a conformational change, which is required for their transient association to secretory carriers, whereas binding to GDP provokes the opposite effect, inducing the release of the Rabs from vesicles membranes (Mizuno-Yamasaki et al., 2012, Wiegandt et al., 2015).

Anterograde traffic of vesicles within the secretory pathway is mainly coordinated by three Rab proteins: Rab1, Rab8, and Rab11 (Hutagalung and Novick, 2011). Rab1 is involved in early events of anterograde transport of vesicles in fungi, plants, insects and humans (Pereira-Leal, 2008, Stenmark, 2012). For instance, Ypt1p, the Rab1 mammalian ortholog in *S. cerevisiae*, is involved in three important steps of the secretory pathway: 1) anterograde traffic of vesicles from ER to early Golgi compartments (Jedd et al., 1997); 2) Intra-Golgi vesicles transport (Suvorova et al., 2002); and 3) early endosomes to late Golgi trafficking (Du and Novick, 2001, Lafourcade et al., 2004, Sclafani et al., 2010). In filamentous fungi, the functions of Ypt1/Rab1 have apparently diversified. In *N. crassa*, YPT-1 was observed at the core of the SPK, coinciding with the spatial distribution of all CHSs, and suggesting its participation in the traffic of secretory vesicles to the hyphal tips (Fig. 4J) (Fajardo-Somera et al., 2015, Riquelme et al., 2007, Sánchez-León et al., 2015, Sánchez-León et al., 2011). A similar localization for RabO was described for *A. nidulans* (Pinar et al., 2013). In addition, co-immunoprecipitation followed by LC/MS of CHS-1, CHS-4, and CHS-5 detected YPT-1 as one of the putative interacting proteins (Fajardo-Somera et al., 2015). Furthermore, YPT-1 sedimented in fractions with a density range similar to the density of fractions with high CHS activity (Bartnicki-Garcia et al., 1984, Leal-Morales et al., 1994, Verdín et al., 2009, Verdín et al., 2015). Together, the evidence points to YPT-1 being the Rab involved in the traffic of chitosomes in *N. crassa*. Whether the traffic of CHS enzymes to the SPK is exclusive to Rab1 orthologs in other fungal systems is still unknown. In *A. nidulans,* the apical recycling of ChsB, the fungal CHS-1 ortholog, is assisted by Rab11 from the TGN (Hernandez-Gonzalez et al., 2018). Although the Rab1 orthologs in *N. crassa* and *A. nidulans* share similarities such as their presence at the SPK and their requirement for cell viability, the stratification of this Rab was identified only in *N. crassa* (Pinar et al., 2013, Sánchez-León et al., 2015). In addition to its apical distribution, YPT-1 was detected co-localizing with *bona fide* markers of the early and late Golgi markers such as USO-1 and SEC-7, respectively. *In vivo* analysis revealed a transient co-localization of YPT-1 with both markers. The dynamic distribution of YPT-1 within Golgi compartments and its conspicuous arrival to the core of the SPK unfolded an additional role of the traffic coordination of this Rab protein that might be essential for the transport of the cell wall nanomachinery.

Studies conducted in the budding yeast have shown that, before being directed to the cell surface from late Golgi, the budding of vesicular carriers is assisted by Ypt31p and Ypt32p, both orthologs of Rab11 (Benli et al., 1996). The membrane association of Ypt31/32p with newly formed post-Golgi vesicles participate in the activation of Sec4p through the recruitment of its GEF factor Sec2p (Ortiz et al., 2002). In *A. nidulans*, the association of its Rab11 ortholog, RabE, at late Golgi cisternae during their maturation process switched its identity into post-Golgi membrane carriers that accumulated at the SPK (Pantazopoulou et al., 2014). *In vivo* localization experiments of YPT-31, the *N. crassa* Rab11 orthologue, revealed the distribution of the fluorescently tagged YPT-31 at the macrovesicular layer of the SPK (Fig. 4K) (Sánchez-León et al., 2015). This particular distribution was confirmed when either the Rab GTPase YPT-1 or the Exocyst subunit EXO-70 tagged with GFP were co-expressed with the tDimer-2-YPT-31. YPT-31 and YPT-1 did not share the same distribution pattern at the SPK, whereas with EXO-70 a partial co-localization was observed (Riquelme et al., 2014, Sánchez-León et al., 2015). This particular localization of YPT-31 at the SPK resembled the spatial arrangement of GS-1 and FKS-1 (Sánchez-León and Riquelme, 2015, Verdín et al., 2009). These observations, although provide only circumstantial evidence, relate this Rab in *N. crassa* with the GSC. In contrast to YPT-1, YPT-31 was not detected at Golgi cisternae, suggesting its exclusive participation in post-Golgi trafficking steps of the secretory vesicles.

Analysis of vesicles dynamics through FRAP experiments revealed that YPT-31 vesicles arrive at very high rates at the hyphal tip (Sánchez-León et al., 2015). The fluorescence recovery of YPT-31 (t_1/2_ 16 s) at the SPK was similar to the recovery rates of Rabs in other fungal systems, whose growth rates are significantly lower compared to *N. crassa* (Jones and Sudbery, 2010, Pantazopoulou et al., 2014). These similarities suggest that the rate of arrival of YPT-31 associated vesicles to the SPK is independent of the hyphal growth rate. One would expect that the rate of arrival of vesicles with cell wall synthesizing components correlates with the hyphal growth rates but based on the above-mentioned FRAP analyses it seems that downstream regulators synchronize the discharge of the vesicles. From earlier studies it has been demonstrated that hyphal elongation rates occur in pulses of growth (López-Franco et al., 1994). Superresolution microscopy analysis in cells of *A. nidulans* revealed that secretory vesicles carrying the Class III ChsB are discharged from the SPK as clusters in an intermittent mode (Zhou et al., 2018).

In *S. cerevisiae*, the last steps of the anterograde traffic of vesicular carriers are linked to the activity of the Rab8 ortholog Sec4p (Salminen and Novick, 1987). Sec4p is distributed at sites of polarized secretion where its interaction with the exocyst complex subunit Sec15p assists in the tethering events crucial for fusion to the plasma membrane (Guo et al., 1999). The subcellular distribution of Sec4 in fungal hyphae has been described elsewhere (Jones and Sudbery, 2010, Pantazopoulou et al., 2014, Powers-Fletcher et al., 2013, Schmitz et al., 2006) and is usually located at the SPK. In *N. crassa* the distribution of SEC-4 was detected at the outer stratum of the SPK resembling the distribution patterns observed for YPT-31, GS-1 and FKS-1 (Fig. 4L) (Sánchez-León et al., 2015, Sánchez-León and Riquelme, 2015, Verdín et al., 2009). This conspicuous allocation of SEC-4 labeled vesicles not only suggests that this Rab protein is associated with the traffic of a specific population of secretory vesicles but also participates in the transport of GSC (Riquelme and Sánchez-León, 2014, Sánchez-León and Riquelme, 2015). Being SEC-4 an effector of YPT-31 (Ortiz et al., 2002) it was not surprising to detect both proteins at the same SPK layer in *N. crassa*. This SEC-4 stratification was not observed in fluorescently tagged Sec4 of other fungal orthologs (Pantazopoulou et al., 2014), despite ultrastructural analyses of hyphal tips that have revealed the presence of at least two populations of vesicles (Hohmann-Marriott et al., 2006). Although further studies are needed to confirm that SEC-4 assists the last traffic steps of the GSC before secretory vesicles are tethered to the target plasma membrane, the evidence so far suggests that in *N. crassa* SEC-4 is involved in this late secretory step of the complex. Further experimental work needs to be performed to elucidate how the Rab proteins coordinate the pre-exocytic events at the SPK at the molecular level.

### 5.5 The exocyst complex

The orchestrated exocytosis of vesicles at active growth regions of the hyphal tips is a crucial step required for successful cell wall biosynthesis (Riquelme et al., 2014). After reaching the hyphal tip and accumulating at the SPK (Bartnicki-García et al., 1995, Riquelme et al., 1998), a tethering process is required to dock the secretory vesicles to specific regions of growth (Caballero-Lima et al., 2013). The tethering of vesicles is assisted by the exocyst, a conserved multimeric protein complex comprised of eight subunits: Sec3, Sec5, Sec6, Sec8, Sec10, Sec15, Exo70 and Exo84 (Guo et al., 1999, TerBush et al., 1996). It has been proposed that the subunits of the complex form two sub-complexes: one subgroup attaches to the membrane of the vesicles, whereas the other is docked to the PM (Wang and Hsu, 2006). However, recent biochemical and structural analyses of the exocyst in *S. cerevisiae* have shown that the complex exists mainly as a stable assembly comprising all eight subunits (Heider et al., 2016). The architecture of the exocyst *in vivo* revealed a putative mechanism of how this tethering complex assists in the contact of membranes between the secretory vesicle and the PM (Picco et al., 2017). The results of recent single-particle Cryo-EM coupled with chemical cross-linking MS analyses has allowed a model for the assembly of the exocyst to be proposed, where the hierarchical interaction of dimeric pairs results in two higher-order structures forming the tetrameric subcomplex I and II (Mei et al., 2018). In particular, the CorEX motif of the Sec3 subunit was found important for recruitment of the other seven subunits and the tethering of secretory vesicles.

Live cell imaging of *N. crassa* hyphae revealed two distribution patterns of the exocyst subunits at the hyphal tips; SEC-5 (NCU07698), -6 (NCU03341), -8 (NCU04190) and -15 (NCU00117) finely extend over the apical PM surface whereas EXO-70 (NCU08012) and EXO-84 (NCU06631) mostly accumulate at the outer layer of the SPK (Fig. 4E–G; Riquelme et al., 2014). SEC-3 (NCU09869) displayed a distribution similar to both of the above-mentioned localization patterns.

In *A. gossypii*, the localization of exocyst components is dependent on the hyphal growth rate (Köhli et al., 2008). AgSec3, AgSec5, and AgExo70 accumulate as a cortical cap at the tip of slow-growing hyphae, whereas they localize at the SPK in fast-growing hyphae. In *C. albicans*, exocyst components Sec3, 6, 8, 15, Exo70, and Exo84 stably localized to an apical crescent (Jones and Sudbery, 2010, Sudbery, 2011). In *A. nidulans*, SECC, the homologue of *S. cerevisiae* Sec3p, was localized in a small region of the apical PM, immediately anterior to the SPK (Taheri-Talesh et al., 2008). In *A. oryzae*, AoSec3 was localized to cortical caps at the hyphal tip as in *A. nidulans* but was also found in septa (Hayakawa et al., 2011). The singularities in the subcellular distribution of the exocyst complex subunits in *N. crassa* and other species suggest specific regulatory mechanisms of the exocyst during the last secretory steps of the cell wall biosynthetic machinery.

The distribution of EXO-70 and EXO-84 subunits at the SPK outer layer in *N. crassa* was similar to that observed for the Rab GTPases YPT-31 and SEC-4, and the GSC (GS-1, FKS-1) (Fig. 4B, C, E, F, K, L) (Riquelme et al., 2014, Sánchez-León et al., 2015, Verdín et al., 2009). This distribution pattern strongly indicates a connection between the exocyst, regulatory proteins and the cell wall synthesis machinery. This preliminary evidence provides hints for upcoming attempts to explore the molecular mechanisms involving both groups of regulators, namely the exocyst and Rab GTPases, in the traffic of cell wall enzymes. The crucial role that the exocyst complex might have in the traffic or fusion of the biosynthetic cell wall machinery was more evident by the drastic morphology defects showed in the studies of *N. crassa sec-5* mutants (Riquelme et al., 2014, Seiler and Plamann, 2003). Analysis of cryo-fixed and freeze-substituted TEM images revealed that hyphae of *sec-5* mutants abnormally accumulated macrovesicles at the hyphal tips indicating a non-functional exocyst complex resulting in an aberrant compact morphology, hyper branching, and the absence of a SPK in FM4-64 stained hyphae. Although there is a correlation between the localization of the complex subunits and the identified cellular and molecular apical apparatus, the differential arrangement of the subunits at apical regions is still intriguing. Is this feature related to the regulation of a specific population of vesicles (i.e. macrovesicles) or does this localization suggest that all the vesicles of the SPK associate with the exocyst components at the macrovesicular area? Further experimental work is still necessary to understand the mechanistic role of the complex at the hyphal apex to gain insights into the cell wall biosynthesis in *N. crassa*.

### 5.6 Transport of proteins involved in cell wall synthesis along the cytoskeleton

During the sophisticated polarized growth of fungal hyphae, the continued expansion of the wall is necessary, and this process requires the coordinated action of the cytoskeleton in the transport of vesicles, proteins and organelles (Riquelme, 2013).

In *N. crassa*, actin localizes at the core of the SPK (Berepiki et al., 2010, Delgado-Álvarez et al., 2010), around the endocytic subapical collar forming small patches, and in association with septum formation (Araujo-Bazán et al., 2008, Berepiki et al., 2010, Delgado-Álvarez et al., 2010, Echauri-Espinosa et al., 2012, Roberson, 1992, Srinivasan et al., 1996, Upadhyay and Shaw, 2008). Actin inhibitor studies using Latrunculin A and Cytochalasin A demonstrated that the actin cytoskeleton is required for the correct localization of CHS-1 (class I CHS) and CHS-4 (class IV CHS), β-1,3-endoglucanases BGT-1 and BGT-2, and GS-1 (Martínez-Nuñez and Riquelme, 2015, Rico-Ramírez et al., 2018, Sánchez-León et al., 2011, Verdín et al., 2009).

CHSs with a MMD (classes V and VII), including CsmA and CsmB of *A. nidulans*, Wdchs5 of *Wangiella dermatitidis,* and Mcs1 of *U. maydis* require the actin cytoskeleton for proper localization (Abramczyk et al., 2009; Takeshita et al., 2005, Treitschke et al., 2010, Tsuizaki et al., 2009). However, the MMD of *A. nidulans* CsmA and CsmB has a role as an anchor to the PM rather than in transport (Takeshita et al., 2005, Takeshita et al., 2006). Similarly, in *U. maydis*, Msc1 MMD is not important for its mobility (Treitschke et al., 2010), which is dependent on myosin-5 and kinesin-1, but instead acts as a tether that supports the fusion of Msc1-carrying vesicles to the PM (Schuster et al., 2012, Treitschke et al., 2010). Moreover, class VII CHSs and GS glucan synthase transport relies on Mcs1 (Schuster et al., 2016).

The microtubule cytoskeleton is important for hyphal morphogenesis and directionality in filamentous fungi (Egan et al., 2012, Fischer et al., 2008, Horio and Oakley, 2005, Konzack et al., 2005, Peñalva, 2010, Riquelme et al., 2000, Riquelme et al., 2002, Seiler et al., 1999, Steinberg, 2011, Xiang and Fischer, 2004). In *N. crassa*, two classes of MT-dependent motors, the minus end-directed dynein and the plus end-directed kinesins, are involved in the positioning of organelles and transport of membranes (Mouriño-Pérez et al., 2016). In *N. crassa*, conventional kinesin-1 KIN-1 (NCU09730) is required for vesicular transport (Seiler et al., 1997). Nevertheless, in *N. crassa Δkin-1* mutants, the stability of CHS-1 at the SPK was affected but not its long range transport (Mouriño-Pérez et al., 2016), suggesting that the MT cytoskeleton is not essential for the delivery of chitosomes.

## 6 Conclusions and future directions

**Fig. 7:**
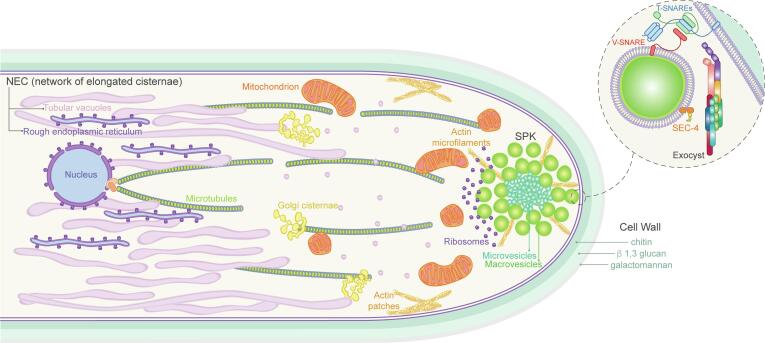
Overall view of the secretory compartments involved in apical cell wall expansion in *N. crassa*. The illustration shows how different organelles including the nucleus, microtubules, NEC (Network of elongated cisternae) comprised by endoplasmic reticulum and tubular vacuoles, and Golgi cisternae, are distributed along the cytoplasm. These compartments work together in the secretion of proteins and vesicles responsible for building the cell wall and allowing cell expansion. The SPK is found at the apex, integrated by secretory vesicles (microvesicles and macrovesicles), actin microfilaments, and ribosomes. The exocyst complex would dock secretory vesicles for further SNARE tethering at sites of polarized growth (dotted circle). The Rab-GTPase (SEC-4) helps to coordinate the tethering process with the vesicle and the acceptor membrane. The cell wall is simply illustrated by three superimposed layers representing chitin, β-1,3 glucan, and galactomannan, respectively.

Significant progress has been made in the last couple of decades relating to the understanding of the molecular and cellular processes that precede the building of the cell wall in *N. crassa* (Fig. 7) and other filamentous fungi. It is quite remarkable that in spite of having analogous cell wall synthetic machineries, fungal species have evolved distinct secretory mechanisms. The structural diversity of the SPK across several fungal taxa is perhaps one of the manifestations of these differences (Dee et al., 2015, Riquelme and Sánchez-León, 2014) and it could be requirements of each fungal species (Köhli et al., 2008). A wide phylogenetic and structural analysis of the fungal secretory machinery is necessary to elucidate the molecular basis of such differences.

The great advancement of microscopy has increased the temporal and spatial resolution of live imaging. Attaining real-time super resolution imaging will be key to elucidate the routes of traffic of vesicles in and out of the SPK along their associated cytoskeletal tracks. Because of their evolutionary implications and consequences in cell wall building and architecture, the differential secretion of the main cell wall synthesizing enzymes, CHS and GSC, needs to be resolved. Moreover, it is necessary to decipher the composition of chitosomes, to determine how many CHS subunits are contained in one chitosome and, more importantly, whether one chitosome contains more than one class of CHS. This information, together with the identification of the mode of activation and inactivation of CHS and the cargo receptors/adaptors for all CHS, will provide a more detailed panorama of the secretion and regulation of these central players in cell wall synthesis.

## Conflict of interest statement

The authors certify that they have NO affiliations with or involvement in any organization or entity with any financial interest (such as honoraria; educational grants; participation in speakers’ bureaus; membership, employment, consultancies, stock ownership, or other equity interest; and expert testimony or patent-licensing arrangements), or non-financial interest (such as personal or professional relationships, affiliations, knowledge or beliefs) in the subject matter or materials discussed in this manuscript.

## References

[b0005] Alcazar-Fuoli L. (2011). Functional analysis of the fungal/plant class chitinase family in *Aspergillus fumigatus*. Fungal Genet Biol..

[b0010] Ao J. (2016). A proteomic and genetic analysis of the *Neurospora crassa* conidia cell wall proteins identifies two glycosyl hydrolases involved in cell wall remodeling. Fungal Genet. Biol..

[b0015] Ao J., Free S.J. (2017). Genetic and biochemical characterization of the GH72 family of cell wall transglycosylases in *Neurospora crassa*. Fungal Genet. Biol..

[b0020] Araujo-Bazán L. (2008). Preferential localization of the endocytic internalization machinery to hyphal tips underlies polarization of the actin cytoskeleton in *Aspergillus nidulans*. Mol. Microbiol..

[b0025] Arroyo J. (2016). Strengthening the fungal cell wall throught chitin-glucan cross-links: effects on morphogenesis and cell integrity. Cell Microbiol..

[b0030] Awald P. (1993). Purification of 1,3-β-glucan synthase from *Neurospora crassa* by product entrapment. Exp. Mycol..

[b0035] Baladron V. (2002). Eng1p, an endo-1,3-beta-glucanase localized at the daughter side of the septum, is involved in cell separation in *Saccharomyces cerevisiae*. Eukaryot. Cell.

[b0040] Bankaitis V.A. (1990). An essential role for a phospholipid transfer protein in yeast Golgi function. Nature.

[b0045] Barlowe C.K., Miller E.A. (2013). Secretory protein biogenesis and traffic in the early secretory pathway. Genetics.

[b0050] Bartnicki-Garcia S. (1968). Cell wall chemistry, morphogenesis, and taxonomy of fungi. Annu. Rev. Microbiol..

[b0055] Bartnicki-Garcia S. (1981). Role of chitosomes in the synthesis of fungal cell walls. Microbiology.

[b0060] Bartnicki-Garcia S. (2006). Chitosomes: past, present and future. FEMS Yeast Res..

[b0065] Bartnicki-García S. (1999). Glucans, walls, and morphogenesis: On the contributions of J.G.H. Wessels to the golden decades of fungal physiology and beyond. Fungal Genet. Biol..

[b0070] Bartnicki-García S., Ashworth J.M., Smith E. (1973). Fundamental aspects of hyphal morphogenesis. Microbial Differentiation.

[b0075] Bartnicki-García S. (1995). Evidence that Spitzenkörper behavior determines the shape of a fungal hypha: a test of the hyphoid model. Exp. Mycol..

[b0080] Bartnicki-Garcia S. (1984). Chitosomes from the wall-less “slime” mutant of *Neurospora crassa*. Arch. Microbiol..

[b0085] Bartnicki-Garcia S., Lippman E. (1969). Fungal morphogenesis: cell wall construction in *Mucor rouxii*. Science.

[b9000] Bartnicki-Garcia S., Lippman E. (1972). Inhibition of *Mucor rouxii* by Polyoxin D: effects on chitin synthetase and morphological development. J. Gen. Microbiol..

[b9005] Bartnicki-Garcia S., Lippman E. (1972). The bursting tendency of hyphal tips of fungi: presumptive evidence for a delicate balance between wall synthesis and wall lysis in apical growth. J. Gen. Microbiol..

[b0090] Basenko E.Y. (2018). FungiDB: an integrated bioinformatic resource fon fungi and oomycetes. J. Fungi.

[b0095] Basmaji F. (2006). The 'interactome' of the Knr4/Smi1, a protein implicated in coordinating cell wall synthesis with bud emergence in *Saccharomyces cerevisiae*. Mol. Genet. Genomics.

[b0100] Beauvais A. (2001). Glucan synthase complex of *Aspergillus fumigatus*. J. Bacteriol..

[b0105] Benli M. (1996). Two GTPase isoforms, Ypt31p and Ypt32p, are essential for Golgi function in yeast. EMBO J..

[b0110] Berepiki A. (2010). F-actin dynamics in *Neurospora crassa*. Eukaryot. Cell.

[b0115] Blanco N. (2012). Crosslinks in the cell wall of budding yeast control morphogenesis at the mother-bud neck. J. Cell. Sci..

[b0120] Blumenthal H.J., Roseman S. (1957). Quantitative estimation of chitin in fungi. J. Bacteriol..

[b0125] Bonifacino J.S., Glick B.S. (2004). The mechanisms of vesicle budding and fusion. Cell.

[b0130] Borkovich K.A. (2004). Lessons from the genome sequence of *Neurospora crassa*: tracing the path from genomic blueprint to multicellular organism. Microbiol. Mol. Biol. Rev..

[b9015] Bowman B.J. (2009). Structure and distribution of organelles and cellular location of calcium transporters in *Neurospora crassa*. Eukaryot. Cell.

[b0135] Bowman B.J. (2015). Characterization of a novel prevacuolar compartment in *Neurospora crassa*. Eukaryot. Cell.

[b0140] Bowman S.M., Free S.J. (2006). The structure and synthesis of the fungal cell wall. Bioessays.

[b0145] Brul S. (1997). The incorporation of mannoproteins in the cell wall of *S. cerevisiae* and filamentous Ascomycetes. Antonie Van Leeuwenhoek.

[b0150] Caballero-Lima D. (2013). The spatial distribution of the exocyst and actin cortical patches is sufficient to organize hyphal tip growth. Eukaryot. Cell.

[b0155] Cabib E. (2009). Two novel techniques for determination of polysaccharide cross-links show that Crh1p and Crh2p attach chitin to both β (1–6)- and β (1–3) glucan in the *Saccharomyces cerevisiae* cell wall. Eukaryot. Cell.

[b0160] Cabib E. (2007). Crh1p and Crh2p are required for the cross-linking of chitin to β (1–6)glucan in the *Saccharomyces cerevisiae* cell wall. Mol. Microbiol..

[b0165] Cantarel B.L. (2009). The carbohydrate active EnZymes database (CAZy): an expert resource for glycogenomics. Nucl. Acids Res..

[b0170] Cappellaro C. (1998). New potential cell wall glucanases of *Saccharomyces cerevisiae* and their involvement in mating. J. Bacteriol..

[b0175] Cardemil L., Pincheira G. (1979). Characterization of the carbohydrate component of fraction I in the *Neurospora crassa* cell wall. J. Bacteriol..

[b0180] Chaffin W.L. (2008). *Candida albicans* cell wall proteins. Microbiol. Mol. Biol. Rev..

[b0185] Chin C.F. (2012). Dependence of Chs2 ER export on dephosphorylation by cytoplasmic Cdc14 ensures that septum formation follows mitosis. Mol. Biol. Cell.

[b0190] Choquer M. (2004). Survey of the *Botrytis cinerea* chitin synthase multigenic family through the analysis of six euascomycetes genomes. Eur. J. Biochem..

[b0195] Cole L. (2000). Brefeldin A affects growth, endoplasmic reticulum, Golgi bodies, tubular vacuole system, and secretory pathway in *Pisolithus tinctorius*. Fungal Genet. Biol..

[b0200] Cortes J.C. (2005). The novel fission yeast (1,3)beta-d-glucan synthase catalytic subunit Bgs4p is essential during both cytokinesis and polarized growth. J. Cell. Sci..

[b0205] Coutinho P.M. (2003). An evolving hierarchical family classification for glycosyltransferases. J. Mol. Biol..

[b0210] Dagkessamanskaia A. (2010). Functional dissection of an intrinsically disordered protein: understanding the roles of different domains of Knr4 protein in protein-protein interactions. Protein Sci..

[b0215] Dagkessamanskaia A. (2010). Knr4 N-terminal domain controls its localization and function during sexual differentiation and vegetative growth. Yeast.

[b0220] Daskalov A. (2017). Molecular mechanisms regulating cell fusion and heterokaryon formation in filamentous fungi. Microbiol. Spectrosc..

[b0225] Davies G., Henrissat B. (1995). Structures and mechanisms of glycosyl hydrolases. Structure.

[b0230] Dee J.M. (2015). Cytology and molecular phylogenetics of Monoblepharidomycetes provide evidence for multiple independent origins of the hyphal habit in the Fungi. Mycologia.

[b0235] De Groot P.W. (2005). Features and functions of covalently linked proteins in fungal cell walls. Fungal Genet. Biol..

[b0240] De Terra N., Tatum E.L. (1961). Colonial growth of *Neurospora*. Sorbose and enzymes alter the composition of the cell wall and induce morphological changes. Science.

[b0245] Dekker N. (2004). Role of the alpha-glucanase Agn1p in fission-yeast cell separation. Mol. Biol. Cell.

[b0250] Delgado-Álvarez D.L. (2010). Visualization of F-actin localization and dynamics with live cell markers in *Neurospora crassa*. Fungal Genet. Biol..

[b0255] DeMarini D.J. (1997). A septin-based hierarchy of proteins required for localized deposition of chitin in the *Saccharomyces cerevisiae* cell wall. J. Cell. Biol..

[b0260] Dharwada S.T. (2018). The chaperone Chs7 forms a stable complex with Chs3 and promotes its activity at the cell surface. Traffic.

[b0265] Du L.L., Novick P. (2001). Yeast rab GTPase-activating protein Gyp1p localizes to the Golgi apparatus and is a negative regulator of Ypt1p. Mol. Biol. Cell.

[b0270] Dueñas-Santero E. (2010). Characterization of glycoside hydrolase family 5 proteins in *Schizosaccharomyces pombe*. Eukaryot. Cell.

[b0275] Duran A. (1975). Chitin synthetase zymogen is attached to the yeast plasma membrane. Proc. Natl. Acad. Sci. U.S.A..

[b0280] Durand F. (2008). Structure-function analysis of Knr4/Smi1, a newly member of intrinsically disordered proteins family, indispensable in the absence of a functional PKC1-SLT2 pathway in *Saccharomyces cerevisiae*. Yeast.

[b0285] Echauri-Espinosa R.O. (2012). Coronin is a component of the endocytic collar of hyphae of *Neurospora crassa* and is necessary for normal growth and morphogenesis. PLoS One.

[b0290] Egan M.J. (2012). Microtubule-based transport in filamentous fungi. Curr. Opin. Microbiol..

[b0295] Encinar del Dedo J. (2009). Beta-glucanase Eng2 is required for ascus wall endolysis after sporulation in the fission yeast *Schizosaccharomyces pombe*. Eukaryot. Cell.

[b0300] Enderlin C.S., Selitrennikoff C.P. (1994). Cloning and characterization of a *Neurospora crassa* gene required for (1,3) beta-glucan synthase activity and cell wall formation. Proc. Natl. Acad. Sci. U.S.A..

[b0305] Esteban P.F. (2005). Characterization of the CaENG1 gene encoding an endo-1,3-beta-glucanase involved in cell separation in *Candida albicans*. Curr. Microbiol..

[b0310] Fajardo-Somera R.A. (2015). Dissecting the function of the different chitin synthases in vegetative growth and sexual development in *Neurospora crassa*. Fungal Genet. Biol..

[b0315] Fischer R. (2008). Polarized growth in fungi-interplay between the cytoskeleton, positional markers and membrane domains. Mol. Microbiol..

[b0320] Fu C. (2014). The *Neurospora crassa* CPS-1 polysaccharide synthase functions in cell wall biosynthesis. Fungal Genet. Biol..

[b0325] Fu C. (2014). *Neurospora crassa* 1,3-alpha-glucan synthase, AGS-1, is required for cell wall biosynthesis during macroconidia development. Microbiology.

[b0330] Gastebois A. (2010). Characterization of a new beta(1–3)-glucan branching activity of *Aspergillus fumigatus*. J. Biol. Chem..

[b0335] Glaser L., Brown D.H. (1957). The synthesis of chitin in cell-free extracts of *Neurospora crassa*. J. Biol. Chem..

[b0340] Gomez-Esquer F. (2004). CRR1, a gene encoding a putative transglycosidase, is required for proper spore wall assembly in *Saccharomyces cerevisiae*. Microbiology.

[b0345] Goncalves I.R. (2016). Genome-wide analyses of chitin synthases identify horizontal gene transfers towards bacteria and allow a robust and unifying classification into fungi. BMC Evol. Biol..

[b0350] Gooday G.W. (1992). What are the roles of chitinases in the growing fungus?. FEMS Microbiol Lett..

[b0355] Grosshans B.L. (2006). The yeast lgl family member Sro7p is an effector of the secretory Rab GTPase Sec4p. J. Cell Biol..

[b0360] Guo W. (1999). The exocyst is an effector for Sec4p, targeting secretory vesicles to sites of exocytosis. EMBO J..

[b0370] Hammond T.M. (2017). Sixteen years of meiotic silencing by unpaired DNA. Adv. Genet..

[b0375] Hartland R.P. (1994). The linkage of (1–3)-beta-glucan to chitin during cell-wall assembly in *Saccharomyces cerevisiae*. Yeast.

[b0380] Hayakawa Y. (2011). Septum-directed secretion in the filamentous fungus *Aspergillus oryzae*. Mol. Microbiol..

[b0385] Heider M.R. (2016). Subunit connectivity, assembly determinants and architecture of the yeast exocyst complex. Nat. Struct. Mol. Biol..

[b0390] Hernandez-Gonzalez M. (2018). Endocytic recycling via the TGN underlies the polarized hyphal mode of life. PLoS Genet..

[b0395] Herold I., Yarden O. (2016). Regulation of *Neurospora crassa* cell wall remodeling via the *cot-1* pathway is mediated by *gul-1*. Curr. Genet..

[b0400] Herve J.C., Bourmeyster N. (2018). Rab GTPases, master controllers of eukaryotic trafficking. Small GTPases.

[b0405] Hickey P.C. (2004). Live-cell imaging of filamentous fungi using vital fluorescent dyes and confocal microscopy. Methods Microbiol..

[b0410] Hohmann-Marriott M.F. (2006). Application of electron tomography to fungal ultrastructure studies. New Phytol..

[b0415] Hong Z. (1994). Cloning and characterization of KNR4, a yeast gene involved in (1,3)-beta-glucan synthesis. Mol. Cell Biol..

[b0420] Horio T., Oakley B.R. (2005). The role of microtubules in rapid hyphal tip growth of *Aspergillus nidulans*. Mol. Biol. Cell.

[b0425] Horn S.J. (2006). Endo/exo mechanism and processivity of family 18 chitinases produced by *Serratia marcescens*. FEBS J..

[b0430] Horsch M. (1997). Beta-N-acetylhexosaminidase: a target for the design of antifungal agents. Pharmacol. Ther..

[b0435] Hrmova M. (1989). 1,3-b-d-Glucan synthase of *Neurospora crasssa*: partial purification and characterization of solubilized enzyme activity. Exp. Mycol..

[b0440] Hunsley D., Gooday G.W. (1974). The structure and development of septa in *Neurospora crassa*. Protoplasma.

[b0445] Hutagalung A.H., Novick P.J. (2011). Role of Rab GTPases in membrane traffic and cell physiology. Physiol. Rev..

[b0450] Ihrmark K. (2010). Comparative molecular evolution of *Trichoderma* chitinases in response to mycoparasitic interactions. Evol. Bioinform. Online.

[b0455] Jedd G. (1997). Two new Ypt GTPases are required for exit from the yeast *trans*-Golgi compartment. J. Cell Biol..

[b0460] Jin Y. (2011). Myosin V transports secretory vesicles via a Rab GTPase cascade and interaction with the exocyst complex. Dev. Cell.

[b0465] Jones L.A., Sudbery P.E. (2010). Spitzenkörper, exocyst, and polarisome components in *Candida albicans* hyphae show different patterns of localization and have distinct dynamic properties. Eukaryot. Cell.

[b0470] Kamei M. (2013). Deletion and expression analysis of beta-(1,3)-glucanosyltransferase genes in *Neurospora crassa*. Fungal Genet. Biol..

[b0475] Karlsson M., Stenlid J. (2008). Comparative evolutionary histories of the fungal chitinase gene family reveal non-random size expansions and contractions due to adaptive natural selection. Evol. Bioinform. Online.

[b0480] Karlsson M., Stenlid J. (2009). Evolution of family 18 glycoside hydrolases: diversity, domain structures and phylogenetic relationships. J. Mol. Microbiol. Biotechnol..

[b0485] King L., Butler G. (1998). Ace2p, a regulator of CTS1 (chitinase) expression, affects *Saccharomyces cerevisiae*. Curr. Genet..

[b0490] Kitagaki H. (2002). Two homologous genes, *DCW1 (YKL046*c) and *DFG*5, are essential for cell growth and encode glycosylphosphatidylinositol (GPI)-anchored membrane proteins required for cell wall biogenesis in *Saccharomyces cerevisia*e. Mol. Microbiol..

[b0495] Köhli M. (2008). Growth-speed-correlated localization of exocyst and polarisome components in growth zones of *Ashbya gossypii* hyphal tips. J. Cell. Sci..

[b0500] Konzack S. (2005). The role of the kinesin motor KipA in microtubule organization and polarized growth of *Aspergillus nidulans*. Mol. Biol. Cell.

[b0505] Kota J., Ljungdahl P.O. (2005). Specialized membrane-localized chaperones prevent aggregation of polytopic proteins in the ER. J. Cell Biol..

[b0510] Kozubowski L. (2003). A Bni4-Glc7 phosphatase complex that recruits chitin synthase to the site of bud emergence. Mol. Biol. Cell.

[b0515] Kuranda M.J., Robbins P.W. (1987). Cloning and heterologous expression of glycosidase genes from *Saccharomyces cerevisiae*. Proc. Natl. Acad. Sci. U.S.A..

[b0520] Kuranda M.J., Robbins P.W. (1991). Chitinase is required for cell separation during growth of *Saccharomyces cerevisiae*. J. Biol. Chem..

[b0525] Lafourcade C. (2004). The GTPase-activating enzyme Gyp1p is required for recycling of internalized membrane material by inactivation of the Rab/Ypt GTPase Ypt1p. Mol. Cell Biol..

[b0530] Lam K.K. (2006). Palmitoylation by the DHHC protein Pfa4 regulates the ER exit of Chs3. J. Cell Biol..

[b0535] Leal-Morales C.A. (1994). Distribution of chitin synthetase and various membrane marker enzymes in chitosomes and other organelles of the slime mutant of *Neurospora crassa*. Exp. Mycol..

[b0540] Leal-Morales C.A. (1988). Localization of chitin synthetase in cell-free homogenates of *Saccharomyces cerevisiae*: chitosomes and plasma membrane. Proc. Natl. Acad. Sci. U.S.A..

[b0545] Leal J.A. (1996). Structural investigation of a cell-wall galactomannan from *Neurospora crassa* and *N. sitophila*. Carbohydr. Res..

[b0550] Lehmler C. (1997). Identification of a motor protein required for filamentous growth in *Ustilago maydis*. EMBO J..

[b0555] Lenardon M.D. (2010). Phosphorylation regulates polarisation of chitin synthesis in *Candida albicans*. J. Cell Sci..

[b0560] Lesage G., Bussey H. (2006). Cell wall assembly in *Saccharomyces cerevisiae*. Microbiol. Mol. Biol. Rev..

[b0565] Lesage G. (2004). Analysis of b-1,3-glucan assembly in *Saccharomyces cerevisiae* using a synthetic interaction network and altered sensitivity to caspofungin. Genetics.

[b0570] Levin D.E. (2005). Cell wall integrity signaling in *Saccharomyces cerevisiae*. Microbiol. Mol. Biol. Rev..

[b0575] Levin D.E. (2011). Regulation of cell wall biogenesis in *Saccharomyces cerevisiae*: the cell wall integrity signaling pathway. Genetics.

[b0580] Lichius A. (2012). Comparative live-cell imaging analyses of SPA-2, BUD-6 and BNI-1 in *Neurospora crassa* reveal novel features of the filamentous fungal polarisome. PLoS One.

[b0585] López-Franco R. (1994). Pulsed growth of fungal hyphal tips. Proc. Natl. Acad. Sci. U.S.A..

[b0590] Maddi A. (2009). Trifluoromethanesulfonic acid-based proteomic analysis of cell wall and secreted proteins of the ascomycetous fungi *Neurospora crassa* and *Candida albicans*. Fungal Genet. Biol..

[b0595] Maddi A. (2012). WSC-1 and HAM-7 are MAK-1 MAP kinase pathway sensors required for cell wall integrity and hyphal fusion in *Neurospora crassa*. PLoS One.

[b0600] Maddi A. (2012). The *Neurospora crassa dfg5* and *dcw1* genes encode alpha-1,6-mannanases that function in the incorporation of glycoproteins into the cell wall. PLoS One.

[b0605] Mahadevan Pr Fau - Mahadkar U.R., Mahadkar U.R. (1970). Role of enzymes in growth and morphology of *Neurospora crassa*: cell-wall-bound enzymes and their possible role in branching. J. Bacteriol..

[b0610] Mahadevan P.R., Tatum E.L. (1965). Relationship of the major constituents of the *Neurospora crassa* cell wall to wild-type and colonial morphology. J. Bacteriol..

[b0615] Mahadevan P.R., Tatum E.L. (1967). Localization of structural polymers in the cell wall of *Neurospora crassa*. J. Cell Biol..

[b0620] Mandel M.A. (2006). *Coccidioides posadasii* contains single chitin synthase genes corresponding to classes I to VII. Fungal Genet. Biol..

[b0625] Manocha M.S., Colvin J.R. (1967). Structure and composition of the cell wall of *Neurospora crassa*. J. Bacteriol..

[b0630] Marchler-Bauer A. (2017). CDD/SPARCLE: functional classification of proteins via subfamily domain architectures. Nucl. Acids Res.

[b0635] Markina-Iñarrairaegui A. (2013). The *Aspergillus nidulans* peripheral ER: disorganization by ER stress and persistence during mitosis. PLoS One.

[b0640] Martin-Yken H. (2003). The interaction of Slt2 MAP kinase with Knr4 is necessary for signalling through the cell wall integrity pathway in *Saccharomyces cerevisiae*. Mol. Microbiol..

[b0645] Martin-Yken H. (2002). KNR4 is a member of the PKC1 signalling pathway and genetically interacts with BCK2, a gene involved in cell cycle progression in *Saccharomyces cerevisiae*. Curr. Genet..

[b0650] Martínez-Nuñez L., Riquelme M. (2015). Role of BGT-1 and BGT-2, two predicted GPI-anchored glycoside hydrolases/glycosyltransferases, in cell wall remodeling in *Neurospora crassa*. Fungal Genet. Biol..

[b0655] Martinez J.P. (1989). Sedimentation properties of chitosomal chitin synthetase from the wild-type strain and the 'slime' variant of *Neurospora crassa*. Biochim. Biophys. Acta.

[b0660] McMurrough I., Bartnicki-Garcia S. (1971). Properties of a particulate chitin synthetase from *Mucor rouxii*. J. Biol. Chem..

[b0665] Mei K. (2018). Cryo-EM structure of the exocyst complex. Nat. Struct. Mol. Biol..

[b0670] Mélida H. (2014). Deciphering the uniqueness of Mucoromycotina cell walls by combining biochemical and phylogenomic approaches. Environ. Microbiol..

[b0675] Mizuno-Yamasaki E. (2012). GTPase networks in membrane traffic. Annu. Rev. Biochem..

[b0680] Mouriño-Pérez R.R. (2016). Microtubules and associated molecular motors in *Neurospora crassa*. Mycologia.

[b0685] Mouyna I. (2000). Glycosylphosphatidylinositol-anchored glucanosyltransferases play an active role in the biosynthesis of the fungal cell wall. J. Biol. Chem..

[b9010] Mouyna I. (2000). Identification of the catalytic residues of the first family of beta(1,3)glucanosyltransferases identified in fungi. Biochem. J..

[b0690] Mouyna I. (2013). Beta-1,3-glucan modifying enzymes in *Aspergillus fumigatus*. Front. Microbiol..

[b0695] Mouyna I. (1998). A 1,3-beta-glucanosyltransferase isolated from the cell wall of *Aspergillus fumigatus* is a homologue of the yeast Bgl2p. Microbiology.

[b0700] Mouyna I. (2004). Gene silencing with RNA interference in the human pathogenic fungus *Aspergillus fumigatus*. FEMS Microbiol. Lett..

[b0705] Mouyna I. (2005). Deletion of GEL2 encoding for a β(1–3)glucanosyltransferase affects morphogenesis and virulence in *Aspergillus fumigatus*. Mol. Microbiol..

[b0710] Mrsa V. (1993). Purification and characterization of the *Saccharomyces cerevisiae* BGL2 gene product, a cell wall endo-beta-1,3-glucanase. J. Bacteriol..

[b0715] Muhlschlegel F.A., Fonzi W.A. (1997). *PHR2* of *Candida albicans* encodes a functional homolog of the pH-regulated gene *PHR1* with an inverted pattern of pH-dependent expression. Mol. Cell Biol..

[b0720] Nakajima T. (1984). Structure of the cell wall proteogalactomannan from *Neurospora crassa*. II. Structural analysis of the polysaccharide part. J. Biochem..

[b0725] Nakajima H. (1991). A cytoskeleton-related gene, uso1, is required for intracellular protein transport in *Saccharomyces cerevisiae*. J. Cell Biol..

[b0730] Nakazawa T. (1998). Isolation and characterization of EPD1, an essential gene for pseudohyphal growth of a dimorphic yeast, *Candida maltosa*. J. Bacteriol..

[b0735] Odronitz F., Kollmar M. (2008). Comparative genomic analysis of the arthropod muscle myosin heavy chain genes allows ancestral gene reconstruction and reveals a new type of 'partially' processed pseudogene. BMC Mol. Biol..

[b0740] Ono N. (2000). The yeast Chs4 protein stimulates the trypsin-sensitive activity of chitin synthase 3 through an apparent protein–protein interaction. Microbiology.

[b0745] Ortiz D. (2002). Ypt32 recruits the Sec4p guanine nucleotide exchange factor, Sec2p, to secretory vesicles; evidence for a Rab cascade in yeast. J. Cell Biol..

[b0750] Pantazopoulou A. (2014). Maturation of late Golgi cisternae into RabE (RAB11) exocytic post-Golgi carriers visualized in vivo. Mol. Biol. Cell.

[b0755] Peñalva M.A. (2010). Endocytosis in filamentous fungi: Cinderella gets her reward. Curr. Opin. Microbiol..

[b0760] Pereira-Leal J.B. (2008). The Ypt/Rab family and the evolution of trafficking in fungi. Traffic.

[b0765] Picco A. (2017). The in vivo architecture of the exocyst provides structural basis for exocytosis. Cell.

[b0770] Pinar M. (2013). Acute inactivation of the *Aspergillus nidulans* Golgi membrane fusion machinery: correlation of apical extension arrest and tip swelling with cisternal disorganization. Mol. Microbiol..

[b0775] Pitson S.M. (1993). Noncellulolytic fungal b-glucanases: their physiology and regulation. Enzyme Microb. Technol..

[b0780] Planas A. (2000). Bacterial 1,3–1,4-l-glucanases: structure, function and protein engineering. Biochim. Biophys. Acta.

[b0785] Powers-Fletcher M.V. (2013). Deletion of the *sec4* homolog *srgA* from *Aspergillus fumigatus* is associated with an impaired stress response, attenuated virulence and phenotypic heterogeneity. PLoS One.

[b0790] Prinz W.A. (2000). Mutants affecting the structure of the cortical endoplasmic reticulum in *Saccharomyces cerevisiae*. J. Cell Biol..

[b0795] Qadota H. (1996). Identification of yeast Rho1p GTPase as a regulatory subunit of 1,3-beta-glucan synthase. Science.

[b0800] Ragni E. (2007). GAS2 and GAS4, a pair of developmentally regulated genes required for spore wall assembly in *Saccharomyces cerevisiae*. Eukaryot. Cell.

[b0805] Ragni E. (2007). The gas family of proteins of *Saccharomyces cerevisiae*: characterization and evolutionary analysis. Yeast.

[b0810] Richards T.A. (2017). What defines the “kingdom” fungi?. Microbiol. Spectrosc..

[b0815] Richthammer C. (2012). RHO1 and RHO2 share partially overlapping functions in the regulation of cell wall integrity and hyphal polarity in *Neurospora crassa*. Mol. Microbiol..

[b0820] Rico-Ramírez A.M. (2018). Imaging the secretory compartments involved in the intracellular traffic of CHS-4, a class IV chitin synthase, in *Neurospora crassa*. Fungal Genet. Biol..

[b0825] Riquelme M. (2013). Tip growth in filamentous fungi: a road trip to the apex. Annu. Rev. Microbiol..

[b0830] Riquelme M. (2018). Fungal morphogenesis, from the polarized growth of hyphae to complex reproduction and infection structures. Microbiol. Mol. Biol. Rev..

[b0835] Riquelme M., Bartnicki-García S. (2008). Advances in understanding hyphal morphogenesis: ontogeny, phylogeny and cellular localization of chitin synthases. Fungal Biol. Rev..

[b0840] Riquelme M. (2007). Spitzenkörper localization and intracellular traffic of green fluorescent protein-labeled CHS-3 and CHS-6 chitin synthases in living hyphae of *Neurospora crassa*. Eukaryot. Cell.

[b0845] Riquelme M. (2014). The *Neurospora crassa* exocyst complex tethers Spitzenkörper vesicles to the apical plasma membrane during polarized growth. Mol. Biol. Cell.

[b0850] Riquelme M. (2000). Dynein and dynactin deficiencies affect the formation and function of the Spitzenkörper and distort hyphal morphogenesis of *Neurospora crassa*. Microbiology.

[b0855] Riquelme M. (1998). What determines growth direction in fungal hyphae?. Fungal Genet. Biol..

[b0860] Riquelme M. (2002). The effects of ropy-1 mutation on cytoplasmic organization and intracellular motility in mature hyphae of *Neurospora crassa*. Fungal Genet. Biol..

[b0865] Riquelme M., Wendland J. (2016). Hyphal tip growth in filamentous fungi. Growth, Differentiation and Sexuality.

[b0870] Riquelme M., Sánchez-León E. (2014). The Spitzenkörper: a choreographer of fungal growth and morphogenesis. Curr. Opin. Microbiol..

[b0875] Riquelme M. (2011). Architecture and development of the Neurospora crassa hypha – a model cell for polarized growth. Fungal Biol..

[b0880] Roberson R. (2012). Ultrastructural locations of chitin synthase in fungal cells of *Neurospora crassa*. Microsc. Microanal..

[b0885] Roberson R.W. (1992). The actin cytoskeleton in hyphal cells of *Sclerotium rolfsii*. Mycologia.

[b0890] Robertson N.F. (1959). Experimental control of hyphal branching and branch form in hyphomycetous fungi. Bot. J. Linn. Soc..

[b0895] Robertson N.F. (1968). The growth process in fungi. Ann Rev Phytopathol..

[b0900] Roche C.M. (2014). *Neurospora crassa*: looking back and looking forward at a model microbe. Am. J. Bot..

[b0905] Rodríguez-Peña J.M. (2000). A novel family of cell wall-related proteins regulated differently during the yeast life cycle. Mol. Cell. Biol..

[b0910] Rolli E. (2011). Expression, stability, and replacement of glucan-remodeling enzymes during developmental transitions in *Saccharomyces cerevisiae*. Mol. Biol. Cell.

[b0915] Rolli E. (2010). GAS3, a developmentally regulated gene, encodes a highly mannosylated and inactive protein of the Gas family of *Saccharomyces cerevisiae*. Yeast.

[b0920] Rose J.K.C. (2002). The XTH family of enzymes involved in xyloglucan endotransglucosylation and endohydrolysis: current perspectives and a new unifying nomenclature. Plant Cell Physiol..

[b0925] Ruiz-Herrera J. (2002). Evolution and phylogenetic relationships of chitin synthases from yeasts and fungi. FEMS Yeast Res..

[b0930] Ruiz-Herrera J. (1975). Microfibril assembly by granules of chitin synthetase. Proc. Natl. Acad. Sci. U.S.A..

[b0935] Salminen A., Novick P.J. (1987). A ras-like protein is required for a post-Golgi event in yeast secretion. Cell.

[b0940] Sanchatjate S., Schekman R. (2006). Chs5/6 complex: a multiprotein complex that interacts with and conveys chitin synthase III from the trans-Golgi network to the cell surface. Mol. Biol. Cell.

[b0945] Sánchez-León E. (2015). The Rab GTPase YPT-1 associates with Golgi cisternae and Spitzenkörper microvesicles in *Neurospora crassa*. Mol. Microbiol..

[b0950] Sánchez-León E., Riquelme M. (2015). Live imaging of beta-1,3-glucan synthase FKS-1 in *Neurospora crassa* hyphae. Fungal Genet. Biol..

[b0955] Sánchez-León E. (2011). Traffic of Chitin Synthase 1 (CHS-1) to the Spitzenkörper and developing septa in hyphae of *Neurospora crassa*: actin dependence and evidence of distinct microvesicle populations. Eukaryot. Cell.

[b0960] Sanz M. (2005). *Candida albicans* strains deficient in CHS7, a key regulator of chitin synthase III, exhibit morphogenetic alterations and attenuated virulence. Microbiology.

[b0965] Sanz M. (2004). *Saccharomyces cerevisiae* Bni4p directs the formation of the chitin ring and also participates in the correct assembly of the septum structure. Microbiology.

[b0970] Saporito-Irwin S.M. (1995). *PHR1,* a pH regulated gene of *Candida albicans*, is required for morphogenesis. Mol. Cell. Biol..

[b0975] Sarthy A.V. (1997). Phenotype in *Candida albicans* of a disruption of the BGL2 gene encoding a 1,3-beta-glucosyltransferase. Microbiology.

[b0980] Saxena I.M. (1995). Multidomain architecture of beta-glycosyl transferases: implications for mechanism of action. J. Bacteriol..

[b0985] Schimoler-O'Rourke R. (2003). *Neurospora crassa* FKS protein binds to the (1,3)beta-glucan synthase substrate, UDP-glucose. Curr. Microbiol..

[b0990] Schmitz H.P. (2006). From function to shape: a novel role of a formin in morphogenesis of the fungus *Ashbya gossypii*. Mol. Biol. Cell.

[b0995] Schnabl M. (2003). Subcellular localization of yeast Sec14 homologues and their involvement in regulation of phospholipid turnover. FEBS J..

[b1000] Schultzhaus Z. (2015). *Aspergillus nidulans* flippase DnfA is cargo of the endocytic collar and plays complementary roles in growth and phosphatidylserine asymmetry with another flippase DnfB. Mol. Microbiol..

[b1005] Schuster M. (2016). Co-delivery of cell-wall-forming enzymes in the same vesicle for coordinated fungal cell wall formation. Nat. Microbiol..

[b1010] Schuster M. (2012). Myosin-5, kinesin-1 and myosin-17 cooperate in secretion of fungal chitin synthase. EMBO J..

[b1015] Sclafani A. (2010). Establishing a role for the GTPase Ypt1p at the late Golgi. Traffic.

[b1020] Segev N. (1988). The yeast GTP-binding YPT1 protein and a mammalian counterpart are associated with the secretion machinery. Cell.

[b1025] Seidl V. (2008). Chitinases of filamentous fungi: a large group of diverse proteins with multiple physiological functions. Fungal Biol. Rev..

[b1030] Seiler S., Plamann M. (2003). The genetic basis of cellular morphogenesis in the filamentous fungus *Neurospora crassa*. Mol. Biol. Cell.

[b9025] Seiler S. (1997). Kinesin is essential for cell morphogenesis and polarized secretion in *Neurospora crassa*. EMBO J..

[b1035] Seiler S. (1999). Kinesin and dynein mutants provide novel insights into the roles of vesicle traffic during cell morphogenesis in *Neurospora*. Curr. Biol..

[b1040] Selker E.U. (1987). Rearrangement of duplicated DNA in specialized cells of *Neurospora*. Cell.

[b1045] Sestak S. (2004). Scw10p, a cell-wall glucanase/transglucosidase important for cell-wall stability in *Saccharomyces cerevisiae*. Microbiology.

[b1050] Sheng W. (2013). Functional differentiation of chitin synthases in *Yarrowia lipolytica*. Biosci. Biotechnol. Biochem..

[b1055] Sogaard M. (1994). A Rab protein is required for the assembly of SNARE complexes in the docking of transport vesicles. Cell.

[b1060] Spatafora J.W. (2017). The Fungal tree of life: from molecular systematics to genome-scale phylogenies. Microbiol. Spectrosc..

[b1065] Srinivasan S. (1996). Functional, organizational, and biochemical analysis of actin in hyphal tip cells of *Allomyces macrogynus*. Mycologia.

[b1070] Stajich J.E. (2017). Fungal genomes and insights into the evolution of the kingdom. Microbiol. Spectrosc..

[b1075] Starr T.L. (2018). The major cellulases CBH-1 and CBH-2 of *Neurospora crassa* rely on distinct ER cargo adaptors for efficient ER-exit. Mol. Microbiol..

[b1080] Starr T.L. (2012). Sorting signals that mediate traffic of chitin synthase III between the TGN/endosomes and to the plasma membrane in yeast. PLoS One.

[b1085] Steinberg G. (2011). Motors in fungal morphogenesis: cooperation versus competition. Curr. Opin. Microbiol..

[b1090] Stenmark H. (2012). The Rabs: a family at the root of metazoan evolution. BMC Biol..

[b1095] Sudbery P. (2011). Fluorescent proteins illuminate the structure and function of the hyphal tip apparatus. Fungal Genet. Biol..

[b1100] Suvorova E.S. (2002). The Sec34/Sec35p complex, a Ypt1p effector required for retrograde intra-Golgi trafficking, interacts with Golgi SNAREs and COPI vesicle coat proteins. J. Cell Biol..

[b1105] Szolderits G. (1989). Membrane properties modulate the activity of a phosphatidylinositol transfer protein from the yeast, *Saccharomyces cerevisiae*. Biochim. Biophys. Acta (BBA)-Biomembr..

[b1110] Taheri-Talesh N. (2008). The tip growth apparatus of *Aspergillus nidulans*. Mol. Biol. Cell.

[b1115] Takeshita N. (2005). CsmA, a class V chitin synthase with a myosin motor-like domain, is localized through direct interaction with the actin cytoskeleton in *Aspergillus nidulans*. Mol. Biol. Cell.

[b1120] Takeshita N. (2006). *Aspergillus nidulans* class V and VI chitin synthases CsmA and CsmB, each with a myosin motor-like domain, perform compensatory functions that are essential for hyphal tip growth. Mol. Microbiol..

[b1125] Teh E.M. (2009). Retention of Chs2p in the ER requires N-terminal CDK1-phosphorylation sites. Cell Cycle.

[b1130] Tentler S. (1997). Inhibition of *Neurospora crassa* growth by a glucan synthase-1 antisense construct. Curr. Microbiol..

[b1135] TerBush D.R. (1996). The Exocyst is a multiprotein complex required for exocytosis in *Saccharomyces cerevisiae*. EMBO J..

[b1140] Thompson J.R. (1999). A glucan synthase FKS1 homolog in *Cryptococcus neoformans* is single copy and encodes an essential function. J. Bacteriol..

[b1145] Tougan T. (2002). Meu10 is required for spore wall maturation in *Schizosaccharomyces pombe*. Genes Cells.

[b1150] Trautwein M. (2006). Arf1p, Chs5p and the ChAPs are required for export of specialized cargo from the Golgi. EMBO J..

[b1155] Treitschke S. (2010). The myosin motor domain of fungal chitin synthase V is dispensable for vesicle motility but required for virulence of the maize pathogen *Ustilago maydis*. Plant Cell.

[b1160] Trilla J.A. (1999). Chs7p, a new protein involved in the control of protein export from the endoplasmic reticulum that is specifically engaged in the regulation of chitin synthesis in *Saccharomyces cerevisiae*. J. Cell Biol..

[b1165] Trinci A.P.J., Collinge A.J. (1975). Hyphal wall growth in *Neurospora crassa* and *Geotrichum candidum*. J. Gen. Microbiol..

[b9020] Tsuizaki M. (2009). Myosin motor-like domain of the class VI chitin synthase CsmB is essential to its functions in *Aspergillus nidulans*. Biosci. Biotechnol. Biochem..

[b1170] Tzelepis G.D. (2012). Functional analysis of glycoside hydrolase family 18 and 20 genes in *Neurospora crassa*. Fungal Genet. Biol..

[b1175] Upadhyay S., Shaw B.D. (2008). The role of actin, fimbrin and endocytosis in growth of hyphae in *Aspergillus nidulans*. Mol. Microbiol..

[b1180] Verdín J. (2009). Functional stratification of the Spitzenkörper of *Neurospora crassa*. Mol. Microbiol..

[b1185] Verdín J. (2015). Density gradient centrifugation for enrichment and identification of gfp-tagged chitosomal microvesicles of filamentous fungi. Bio-protocol.

[b9030] Vermeulen C.A., Wessels J.G. (1986). Chitin biosynthesis by a fungal membrane preparation. Evidence for a transient non-crystalline state of chitin. Eur. J. Biochem..

[b1190] VerPlank L., Li R. (2005). Cell cycle-regulated trafficking of Chs2 controls actomyosin ring stability during cytokinesis. Mol. Biol. Cell.

[b1195] Vogt N., Seiler S. (2008). The RHO1-specific GTPase-activating protein LRG1 regulates polar tip growth in parallel to Ndr kinase signaling in *Neurospora*. Mol. Biol. Cell.

[b1200] Wang C.W. (2006). Exomer: a coat complex for transport of select membrane proteins from the trans-Golgi network to the plasma membrane in yeast. J. Cell Biol..

[b1205] Wang S., Hsu S.C. (2006). The molecular mechanisms of the mammalian exocyst complex in exocytosis. Biochem. Soc. Trans..

[b1210] Weber I. (2006). Polar localizing class V myosin chitin synthases are essential during early plant infection in the plant pathogenic fungus *Ustilago maydis*. Plant Cell.

[b1215] Wedlich-Söldner R. (2002). Dynein supports motility of endoplasmic reticulum in the fungus *Ustilago maydis*. Mol. Biol. Cell.

[b1220] Weiner A. (2019). On-site secretory vesicle delivery drives filamentous growth in the fungal pathogen *Candida albicans*. Cell Microbiol..

[b1225] Wessels J.G.H. (1988). A steady-state model for apical wall growth in fungi. Acta Bot. Neerl..

[b1230] Wiegandt D. (2015). Locking GTPases covalently in their functional states. Nat. Commun..

[b1235] Xiang X., Fischer R. (2004). Nuclear migration and positioning in filamentous fungi. Fungal Genet. Biol..

[b1240] Yamazaki H. (2008). *Aspergillus nidulans* ChiA is a glycosylphosphatidylinositol (GPI)-anchored chitinase specifically localized at polarized growth sites. Fungal Genet. Biol..

[b1245] Zhang G. (2006). Exit from mitosis triggers Chs2p transport from the endoplasmic reticulum to mother-daughter neck via the secretory pathway in budding yeast. J. Cell Biol..

[b1250] Zhou L. (2018). Superresolution and pulse-chase imaging reveal the role of vesicle transport in polar growth of fungal cells. Sci. Adv..

